# Proteinuria as an independent predictor of stroke: Systematic review and meta-analysis

**DOI:** 10.1177/1747493019895206

**Published:** 2020-01-14

**Authors:** Dearbhla M Kelly, Peter M Rothwell

**Affiliations:** Center for Prevention of Stroke and Dementia, Nuffield Department of Clinical Neurosciences, John Radcliffe Hospital, University of Oxford, Oxford, UK

**Keywords:** Chronic kidney disease, stroke, hypertension, albuminuria, proteinuria, glomerular filtration rate

## Abstract

**Background:**

Proteinuria has emerged as an important vascular risk factor for adverse cardiovascular events including stroke. Hypertension has been proposed as the principal confounder of this relationship but its role has not been systematically examined.

**Aim:**

We aimed to determine if proteinuria remains an independent predictor of stroke after more complete adjustment for blood pressure.

**Summary of review:**

We performed a systematic review, searching MEDLINE and EMBASE (to February 2018) for cohort studies or randomized controlled trials that reported stroke incidence in adults according to baseline proteinuria ± glomerular filtration rate. Study and participant characteristics and relative risks were extracted. Estimates were combined using a random effects model. Heterogeneity was assessed by χ^2^ statistics and I^2^, and by subgroup strata and meta-regression, with a particular focus on the impact of more complete adjustment for blood pressure on the association. The quality of cohort studies and post hoc analyses was assessed using the Newcastle–Ottawa Scale. We identified 38 studies comprising 1,735,390 participants with 26,405 stroke events. Overall, the presence of any level of proteinuria was associated with greater stroke risk (18 studies; pooled crude relative risk 2.00, 95%CI 1.63–2.46; p < 0.001) even after adjustment for established cardiovascular risk factors (33 studies; pooled adjusted relative risk 1.72, 1.51–1.95; p < 0.001), albeit with considerable heterogeneity between studies (p < 0.001; I^2 ^= 77.3%). Moreover, the association did not substantially attenuate with more thorough adjustment for hypertension: single baseline blood pressure measure (10 studies; pooled adjusted relative risk = 1.92, 1.39–2.66; p < 0.001); history or treated hypertension (four studies; pooled adjusted relative risk = 1.76, 1.13–2.75, p = 0.013); multiple blood pressure measurements over months to years (four studies; relative risk = 1.68, 1.33–2.14; p < 0.001).

**Conclusions:**

Even after extensive adjustment for hypertension, proteinuria is strongly and independently associated with incident stroke risk, possibly indicating a shared renal and cerebral susceptibility to vascular injury that is not fully explained by traditional vascular risk factors.

## Introduction

Proteinuria has emerged as an important vascular risk factor for adverse cardiovascular events across a range of populations.^1–3^ It has been suggested that proteinuria not only reflects glomerular damage, but also is a sensitive indicator of generalized endothelial dysfunction and capillary vasculopathy that allows penetration of atherosclerotic lipoproteins into vessel walls.^4^

There appears to be a particularly strong association between proteinuria and stroke. In a previous meta-analysis of 10 cohort studies (140,231 participants; 3266 strokes), participants with proteinuria had a 71% greater risk of stroke compared with those without proteinuria.^5^ The excess risk of stroke also remained significant after adjustment for other vascular risk factors (relative risk (RR) = 1.63). In a larger meta-analysis of 83 studies (over two million participants), a 25 mg/mmol increase in albumin–creatinine ratio was associated with a 10% increased risk of stroke.^6^ Stroke risk increased linearly and additively with declining glomerular filtration rate (GFR) and increasing albuminuria. However, in our recently published systematic review and meta-analysis of low GFR and stroke risk,^7^ we showed that this risk association was greatly attenuated by adjustment for long-term blood pressure (BP) burden, suggesting that hypertension might confound the association.

The “strain vessel hypothesis” has been proposed as a possible mechanism for the relationship between renal and cerebrovascular diseases.^8^ In the kidney, the juxtamedullary afferent arterioles are small and short vessels that have to maintain a strong vascular tone in order to provide a large pressure gradient in a short distance.^9^ These types of vessels are therefore referred to as “strain vessels” as they are most susceptible to hypertensive renal injury with loss of their autoregulatory ability, resulting in glomerular hypertension, sclerosis, and subsequently microalbuminuria.^10^ Cerebral “strain vessels” refer to the deep perforating arteries that arise directly from large high-pressure arteries, such as anterior, middle, or posterior cerebral arteries that also have to maintain large pressure gradients from their parent arteries to brain tissue capillaries.^11^ The areas of blood supply governed by these perforating arteries are frequently the sites of ischemic or hemorrhagic stroke when cerebral autoregulation is impaired by chronic hypertension.^12^ Thus, “strain vessel injuries” mediated by hypertension may explain the link between vascular damage and microalbuminuria in the kidney and cerebrovascular diseases in the brain.

We hypothesize then that any association between proteinuria and stroke risk may therefore be greatly diminished with adequate adjustment for longer-term BP or prior hypertension. Using a systematic review and meta-analysis, we aimed to assess the impact of proteinuria on stroke risk and whether any association present remained after more complete adjustment for BP.

## Methods

### Data sources and searches

Using the same protocol, we updated an earlier systematic review and meta-analysis of randomized controlled trials and cohort studies that had estimated the association between proteinuria and the risk of stroke.^6^ The study protocol was registered prospectively on PROSPERO (CRD42019127301) and conformed with PRISMA guidelines.^13^ We searched MEDLINE (2013–February 2018) and EMBASE (2013–February 2018) databases using a search strategy developed by a specialized librarian that combined text word and medical subject headings without language restrictions (Supplementary Materials, Appendix Table I).

### Study selection

We included all RCTs and cohort studies that measured proteinuria at baseline and reported quantitative estimates with a measure of precision (or original data which allowed their calculation) of the risk of incident or recurrent stroke. Proteinuria must have been quantified by 24 h urine collection, urine aliquot albumin:creatinine ratio or urine protein:creatinine ratio (UPCR), urine dipstick, or agglutination assay. The pre-specified definitions of proteinuria are listed in Table 1. However, studies using equivalent or sex-specific cut-off points were also included. Where data on baseline estimated glomerular filtration rate (eGFR) were available, GFR had to be either estimated using a validated formula (Cockcroft–Gault, modification of diet in renal disease (MDRD), CKD epidemiology collaboration (CKD-EPI)), measured directly, approximated from urinary creatinine clearance or estimable from serum creatinine. The outcome of interest was symptomatic stroke confirmed by physician examination, hospital record review, or identified from data-linkage of administrative records. Eligible articles were evaluated for overlap based on geographical setting, study period, sample size, and outcome. We excluded cross-sectional and case–control studies, studies where albuminuria or GFR was measured using non-validated methods, studies that had mostly participants with end stage renal disease (by history of dialysis or an eGFR <15 ml/min/1.73 m^2^), studies where outcomes were measured by self-reports or proxy reports, and studies that reported radiological but clinically silent stroke disease. Studies that used slightly varying eGFR intervals were included if they were otherwise comparable. Disagreement over eligibility was resolved by discussion with the senior author (PMR).

**Table 1.** Pre-specified definitions of albuminuria categories

Measurement method	Microalbuminuria	Macroalbuminuria
24 h urine collection (mg/d)	30–300	>300
Urine albumin excretion rate	30–300 mg/day or 20–200 µg/min	>300 mg/day or >200 µg/min
Spot urine albumin:creatinine ratio (mg/g, mg/mmol)	30–300, 3.4–34	>300, >34
Spot urine protein:creatinine ratio (mg/g, mg/mmol)	N/A	>300, >45
Spot urine dipstick	N/A	≥1+

N/A: not applicable.

### Data abstraction and quality assessment

Key descriptive and quantitative data were recorded for study characteristics, participants, exposures, and outcomes. We collected details of the year of study publication, location, size, and duration. Abstracted participant characteristics included age, gender, race and the prevalence of diabetes, known vascular diseases, smoking, and hypertension. We also noted if participants were recruited at a time of high stroke risk including around an acute coronary event, coronary revascularization procedure, or carotid arterial intervention. We recorded the quantity of albuminuria, the method of measurement, and the units of quantification used. We then extracted data for the RR, odds ratio or hazard ratio of stroke associated with each quantity of albuminuria (and with each specified eGFR category if available) and noted whether reported strokes were fatal or non-fatal, incident or recurrent, as well as the subtype of stroke (hemorrhagic, ischemic, or unspecified). We obtained effect estimates from both the unadjusted (or minimally adjusted) and the most fully adjusted model presented noting which variables the model had adjusted for. The standard error of the estimate was also extracted or estimated from the reported 95%CI. We assessed the quality of cohort studies and post hoc analyses of trials using the Newcastle–Ottawa Scale.^14^

### Statistical analysis

The leading outcome of interest was the risk of stroke in patients with any level of proteinuria and according to categories (microalbuminuria, macroalbuminuria). We also performed an analysis of studies that reported risk in patients with varying levels of both albuminuria and low eGFR (defined as <60 ml/min/1.72 m^2^). When articles provided estimates based on both the MDRD and CKD-EPI equations, we used estimates from the CKD-EPI equation as these result in more accurate risk prediction for adverse outcomes compared with the MDRD study equation.^15^ We converted RRs associated with the presence or categories of albuminuria to their natural logarithms and synthesized log RRs and standard errors using the DerSimonian and Laird method in a random effects model. A fixed effect model was also used for comparison with the random effects model on the overall risk estimate. Reported P values were two sided, with significance set at less than 0.05. When studies published more than one estimate of the association between proteinuria and risk of stroke according to subtypes (e.g. by gender or type of diabetes), a within-study summary estimate was obtained. Heterogeneity among included studies was assessed by χ^2^ statistics and the I^2^ test. Based on the suggestion of the Cochrane Collaboration we regarded heterogeneity as possibly unimportant when the I^2^ value was less than 40% and considerable when more than 75%.^16^ We used subgroup analyses and meta-regression to explore sources of inconsistency and heterogeneity. Subgroups were pre-specified and included study characteristics (study design, size, location, duration of follow-up), participant characteristics (age, gender, race, prevalence of diabetes, hypertension, smoking, atrial fibrillation, undergoing cardiac or carotid intervention, quantity of albuminuria, eGFR defined by CKD stage), and characteristics of stroke recorded (subtype, severity, and whether incident or recurrent). To evaluate the impact of the type of hypertension adjustment on effect estimates, we derived and reported results according to a hierarchy of adjustment. We proposed the following hierarchy from least adjustment to best: no adjustment, adjustment for single (or few) BP readings at study entry alone, composite adjustment for historical or treated hypertension or single BP measurement, adjustment for only historical or treated hypertension, and adjustment for multiple BP readings over time. We did not specify a single numeric definition of hypertension as the definition of hypertension has clearly changed over time and can differ between guidelines.^17^ We also conducted a sensitivity analysis including only studies that specifically adjusted for angiotensin converting enzyme inhibitor (ACEi) or angiotensin receptor blocker (ARB) use to determine if this treatment mitigated stroke risk. Publication bias was assessed by visual examination of funnel plots. For all analyses, we used Stata software version 13 (Stat Corp., College Station, TX).

## Results

**Figure 1.**
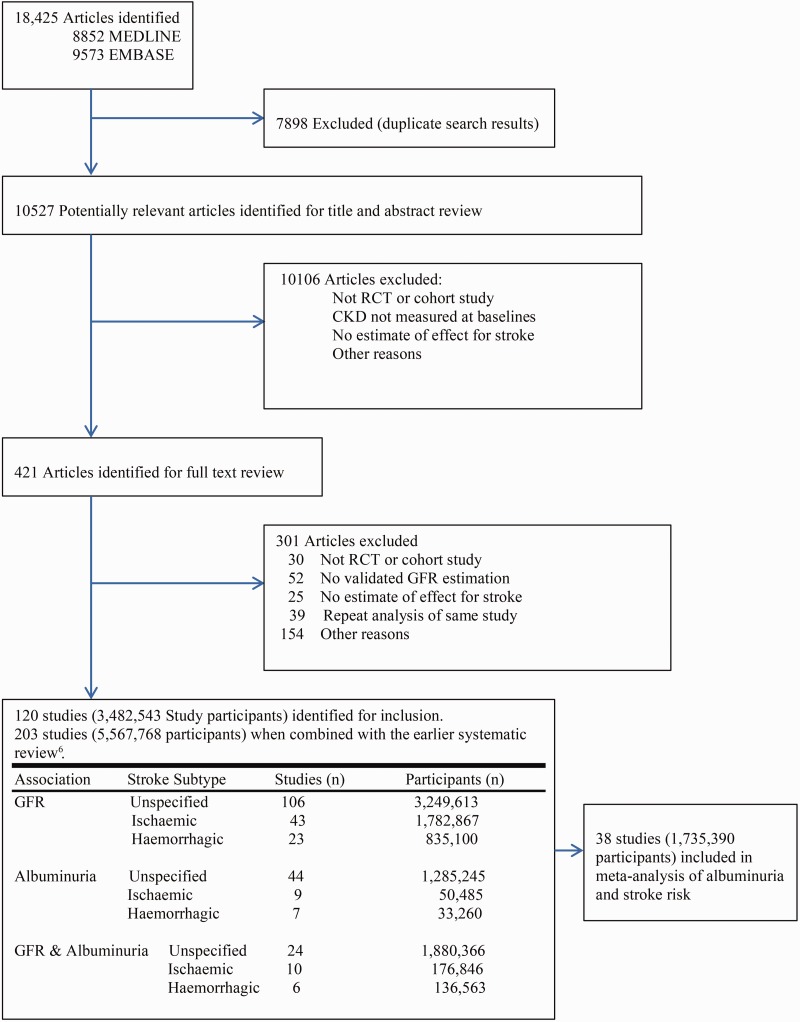
Identification and inclusion of study reports of proteinuria and stroke risk. CKD: Chronic kidney disease; GFR: glomerular filtration rate; RCT: Randomized controlled trial.

Combining with the earlier review of 83 studies,^6^ we identified a further 120 studies that assessed stroke risk in CKD, resulting in 203 studies reporting 118,851 strokes in 5,567,768 participants. Thirty-eight studies (1,735,390 participants) provided appropriate quantitative data to be included in the meta-analysis of proteinuria and stroke risk (Figure 1 and Supplementary Materials, Appendix Table II). The data were derived from six randomized controlled trials and 32 cohort studies. In total, there were 26,405 stroke events including 21,853 which were not classified by pathological subtype (unspecified), 3730 ischemic, and 822 hemorrhagic strokes. Characteristics of the included studies and trials are described in Supplementary Materials, Appendix Tables II and III. The participant number ranged from 295 to 1,023,686. The follow-up duration ranged from 1 to 324 months. Thirty studies (78.9%) reported unspecified stroke types, 14 (36.8%) reported ischemic strokes, and 10 (26.3%) reported hemorrhagic strokes (Supplementary Materials, Appendix Table III). Proteinuria was most commonly quantified using either urine dipstick (14 studies; 36.8%) or urine albumin:creatinine ratio (14 studies; 36.8%) followed by multiple methods (four studies; 10.5%), urine albumin excretion rate (two studies; 5.3%), 24 h urine collection (two studies: 5.3%), and UPCR (one study; 2.6%). The majority of studies were based in North America (12 studies; 31.6%) followed by Asia (10 studies; 26.3%). 36.8% of included studies reported a high prevalence of diabetes (≥30%). Most studies (35/38) were of good quality and provided multivariate adjusted risk estimates (Supplementary Materials, Appendix Table IV).

**Figure 2.**
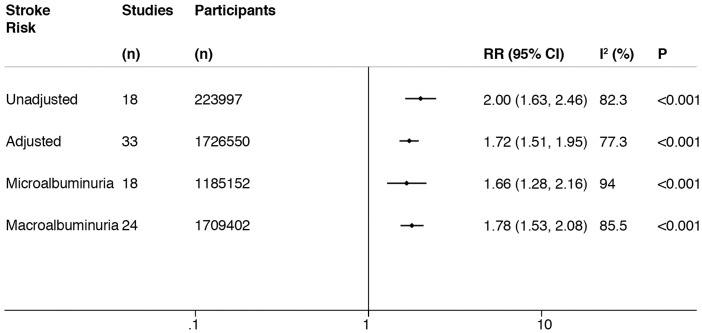
Relative risk (RR) for the association of proteinuria and stroke in unadjusted analysis, in multivariate adjusted analysis that adjusted for conventional vascular risk factors, and in adjusted analysis according to the level of albuminuria. Study and participant numbers along with the level of heterogeneity (I^2^ and P value) also described.

**Figure 3.**
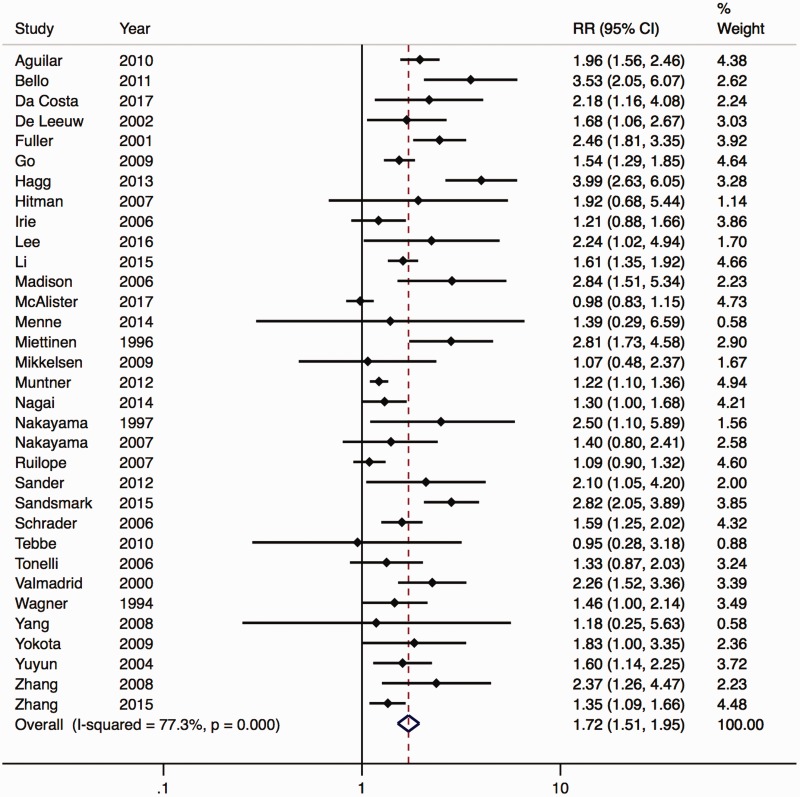
Relative risk (RR) for the association of proteinuria and stroke adjusted for traditional cardiovascular risk factors (exact methods varied between studies).

Pooling unadjusted results from the random effects model showed that incident stroke increased among patients with any level of proteinuria (RR = 2.00, 95%CI 1.63–2.46; p < 0.001) (Figure 2 and Supplementary Materials, Appendix Figure I). In pooled multivariate adjusted analysis, this risk association attenuated to an RR of 1.72 (1.51–1.95; p < 0.001) (Figure 3).

Significant heterogeneity existed between studies (p < 0.001, I^2 ^= 77.3%). The size of the estimate was reduced in a fixed effects model but similar when the analysis was confined to paired studies only (Supplementary Materials, Appendix Figures II and III). From the studies that reported specific stroke subtypes, there was a similar association with ischemic (adjusted RR = 1.99, 1.48–1.66; p < 0.001) and hemorrhagic (adjusted RR = 2.08, 1.65–2.63; p < 0.001) stroke risk (Supplementary Materials, Appendix Figure IV).

Eighteen studies (1,181,884 participants) and 24 studies (1,709,402 participants) reported estimates of the association between microalbuminuria and macroalbuminuria, respectively, with stroke risk. In adjusted-analysis, stroke risk was similar for both macroalbuminuria (adjusted RR = 1.78, 1.53–2.08) and microalbuminuria (adjusted RR = 1.66, 1.28–2.16) (Figure 2 and Supplementary Materials, Appendix Figure V).

The presence of proteinuria was associated with an increased risk of subsequent stroke in all subgroups when estimates were stratified by study design, location, size, quality, duration of follow-up, GFR formula used, mean age groups, gender, race, percentage of diabetics/hypertensives/atrial fibrillation/smokers, setting of high-risk procedure, and stroke type (Supplementary Materials, Appendix Table V). Significant heterogeneity between pooled analyses in univariate meta-regression was noted for smaller studies (<5000 participants) compared with larger studies (>5000 participants) (adjusted RR = 2.05, 1.78–2.37 versus RR = 1.40, 1.22–1.60, P for heterogeneity among subgroups = 0.002) and for studies with younger populations (<60 years) compared with those with older ones (≥60 years) (adjusted RR = 2.12, 1.65–2.70 versus RR = 1.50, 1.32–1.70, P for heterogeneity = 0.02). In addition, there was a 53% lower risk of stroke reported in randomized trials compared to cohort studies (P for heterogeneity = 0.12) and a 51% greater risk in studies that quantified albuminuria using laboratory methods when compared to those that used only urine dipstick (P for heterogeneity = 0.06). We also observed a stronger association with stroke risk in studies with higher prevalence rates of diabetes (≥30%) (adjusted RR = 2.18, 1.60–2.99, P value for heterogeneity = 0.08). Risk of stroke did not vary substantially or consistently by any other study, participant, or stroke characteristic. No variable contributed significantly to heterogeneity in a multivariate meta-regression model. In a sensitivity analysis of three studies that specifically adjusted for ACEi/ARB use (along with other covariates), the presence of proteinuria was still associated with a significantly increased stroke risk (RR = 1.50, 1.05–2.13; p = 0.02).

Seven studies (1,072,129 participants) provided data on the interaction between proteinuria and eGFR. In the presence of a low eGFR, the addition of any level of proteinuria substantially increased the risk of stroke in adjusted analysis (RR = 2.23, 1.48–3.37; p < 0.01) (Supplementary Materials, Appendix Figure VI).

The funnel plot showed some asymmetry consistent with publication bias with smaller studies showing an exaggerated stroke risk association with proteinuria (Supplementary data, Appendix Figure VII). Egger’s test confirms the presence of small-study effects in adjusted analysis (P = 0.003).

**Figure 4.**
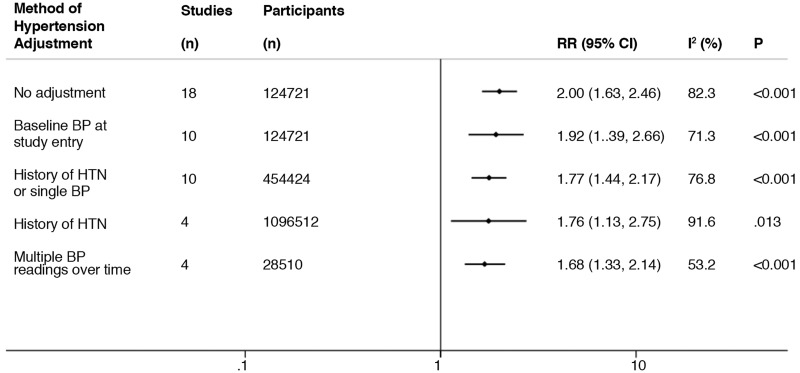
Variation in the Relative risk (RR) for the association of proteinuria and stroke risk depending on the method of hypertension adjustment used in the studies. All studies were also adjusted for other traditional risk factors. Study and participant numbers along with the level of heterogeneity (I^2^ and P value) also described. BP: blood pressure; HTN: hypertension.

As we have previously reported in a meta-analysis of low eGFR and stroke risk,^7^ our proposed hierarchy of methods for hypertension adjustment in the included studies is outlined in Supplementary Materials, Appendix Table VI. There was a downward attenuation of the risk association of proteinuria and stroke after crude analysis with adjustment for a single or few BP readings at study entry resulting in an RR of 1.92 (1.39–2.66) followed by adjustment on the basis of a history of or treated hypertension or a baseline BP reading (a composite definition) (RR = 1.77, 1.44–2.17). Adjusting for only historical or treated hypertension had a similar impact on the relationship as a more composite definition (RR = 1.76, 1.13–2.75). The risk association did not substantially attenuate when only studies adjusting for multiple prior BP readings were included (RR 1.68, 1.33–2.14) (Figure 4).

## Discussion

In this meta-analysis of nearly two million participants with over 26,000 stroke events, we found that the presence of proteinuria confers about a 70% greater risk of stroke compared to that in those without it. This association was consistent across various subgroups and stroke subtypes in the presence of adjustment for cardiovascular risk factors. Although residual confounding may remain, it would appear that albuminuria is an important and likely independent risk factor for stroke. The magnitude of our stroke risk estimate with proteinuria is very consistent with that of an earlier, smaller meta-analysis of 10 studies (140,231 participants; RR = 1.71).^5^

However, the exact mechanism mediating the relationship between proteinuria and stroke is not clear. Linked to ischemic electrocardiographic changes,^18^ carotid artery intima media thickness,^19^ and left ventricular hypertrophy,^20^ albuminuria is thought to be a surrogate marker of subclinical vascular disease and atherosclerosis.^21^ The Steno hypothesis suggests that urinary protein excretion not only reflects localized subclinical renal disease but also a more generalized vascular endothelial dysfunction.^22^ Although albuminuria may reflect generalized endothelial dysfunction, there may also be more specific hemodynamic mechanisms underlying these associations. In the strain vessel hypothesis,^8^ BP has been proposed as the underlying link and we attempted to examine its impact in a sensitivity analysis where we categorized studies according to the extent of their adjustment for hypertension and determined the differential risk associations. Although few studies, even in those that extensively adjusted for prior BP, the effect of proteinuria on stroke risk remained suggesting an independent relationship. This contrasts with our previous meta-analysis of low eGFR and stroke risk where the risk association was greatly attenuated when the analysis was confined to studies that adjusted for multiple prior BPs.^7^ Our results do align with those of previous cohort studies and meta-analyses that have suggested that proteinuria is better predictive of cardiovascular and cerebrovascular events than eGFR.^23,24^

Albuminuria is clearly a marker of stroke risk and post hoc analysis of large-scale clinical trials suggests that it is also a potential target for risk reduction with anti-proteinuric agents. In the Losartan Intervention for Endpoint Reduction in Hypertension (LIFE) study, of 8200 patients with hypertension and left ventricular hypertrophy, losartan therapy reduced occurrence of the primary composite endpoint including death, myocardial infarction, and stroke in both diabetic and non-diabetic participants concomitant with reduction in albuminuria.^25^ In this study, a major part of the risk predicted by values of albuminuria during treatment was also not explained by the level of systolic BP.

Mechanistically it is unclear whether the associations of albuminuria pathways with stroke reflect a causal relationship or mere correlation due to parallel changes in BP or some other similar pathophysiologic process (e.g. generalized endothelial dysfunction). It is likely that the potentially plausible explanations are not mutually exclusive but could act concurrently. From a genome-wide association study of albuminuria in UK Biobank, using Mendelian randomization, a genetic risk score of up to 46 albuminuria variants was strongly associated with increased risk of hypertension and stroke.^26^ There are clearly bidirectional effects between albuminuria and BP that may contribute to stroke risk but our analysis would suggest that there are other important etiological factors outside of this complex interplay.

A shared genetic susceptibility for premature vascular disease may explain the higher stroke risk that we observed in studies of younger populations. There is an extension of the Steno hypothesis^27^ proposing that the level and the inter-individual variation of albuminuria in early life reflect differential vascular states and that one may be endowed at birth with a level of albuminuria that may represent a high-risk vascular state associated with increased susceptibility to organ damage in later life.

There are several potential limitations of this meta-analysis. First, there was only one reviewer and thus the results may be biased if the selection criteria for including a study were applied in a subjective manner. To reduce this risk, any questionable studies for inclusion were discussed with the senior author (PMR). Second, there was considerable heterogeneity in the magnitude of the association between albuminuria and stroke risk across studies. However, although there was significant quantitative heterogeneity, there was remarkably little qualitative heterogeneity. The associations may vary in strength but are almost always in the same direction. Meta-regression analysis did also identify certain characteristics of the studies and study populations that may explain the level of heterogeneity. Some of it may be attributable to study size with greater associations between proteinuria and stroke risk described in smaller studies. Small-study effects could reflect poor methodological quality and lead to an overestimation of the risk estimate, although generally the quality of included studies in this meta-analysis was good when rated using the previously described tools. The mean age of study participants also contributed significantly to heterogeneity, indicating that there may be residual confounding in younger populations such as other renal vascular or genetic risk factors that have not been adjusted for, which is consistent with findings from our prior meta-analysis of low eGFR and stroke risk.^7^ The method of urinary protein quantification may also have been a source of heterogeneity. Sensitivity analysis according to method of albuminuria was undertaken to avoid potential misinterpretation of findings due to assessment bias since urine dipstick measurements have a lower diagnostic accuracy than the other described methods.^28^ In keeping with earlier reviews,^5^ the risk estimate was attenuated in studies that used dipstick testing as the method of measurement, and given that this is an imprecise method of measurement, there may have been regression dilution bias and these studies have likely underestimated the strength of the association between albuminuria and stroke. Indeed, two of the studies^29,30^ contributing most to heterogeneity based their risk estimates on dipstick or mixed method urinary protein quantification. Third, it is challenging to disentangle the potential confounding effects of comorbidities and since this meta-analysis was performed using study-level data as opposed to using individual patient-level data, we were unable to apply a uniform adjustment for confounding variables to all studies which may have led to an overestimation of the true association.

Our meta-analysis supports the hypothesis that proteinuria is an independent risk factor for stroke. This association remained even after adjustment for established cardiovascular risk factors including long-term prior BP. Our findings would therefore tend to refute the “strain vessel hypothesis”^8^ that hypertensive vascular damage completely underpins the relationship between proteinuria and cerebrovascular disease. There may be a causal relationship between pathways leading to albuminuria, likely at a genetic level, and subsequent stroke risk. Proteinuria could be routinely screened for when cardiovascular risk profiling and may need to be incorporated in risk prediction scores.

## Supplemental Material

WSO895206 Supplemental Material - Supplemental material for Proteinuria as an independent predictor of stroke: Systematic review and meta-analysis Click here for additional data file. Supplemental material, WSO895206 Supplemental Material for Proteinuria as an independent predictor of stroke: Systematic review and meta-analysis by Dearbhla M Kelly and Peter M Rothwell in International Journal of Stroke
