# Supplementary material for: Proteinuria as an independent predictor of stroke: Systematic review and meta-analysis
Source: Int J Stroke. 2020 Jan 14;15(1):29–38. doi: 10.1177/1747493019895206 (PMC7003151; doi:10.1177/1747493019895206)
Supplement: WSO895206 Supplemental Material - Supplemental material for Proteinuria as an independent predictor of stroke: Systematic review and meta-analysis [file WSO895206_Supplemental_Material.pdf]

SUPPLEMENTARY MATERIALS

APPENDIX

Proteinuria as an Independent Predictor of Stroke: Systematic Review and Meta-analysis.

Abbreviations:

AAA, abdominal aortic aneurysm; ACE, Angiotensin Converting Enzyme inhibitor; ACR, albumin:creatinine ratio; AF, atrial fibrillation; AR, aortic regurgitation; A2RB, Angiotensin 2 Receptor Blocker; BMI, body mass index; BMS, bare metal stent; Ca, calcium; CABG, coronary artery bypass grafting; CAD, coronary artery disease; CCF, congestive cardiac failure; CEA, carotid endarterectomy; CLD, chronic liver disease; COPD, chronic obstructive pulmonary disease; CRP, C Reactive Protein; CVA, cerebrovascular accident; CVD, cardiovascular disease; DAPT, dual anti-platelet therapy; DBP, diastolic blood pressure; DM, diabetes mellitus; ECG, electrocardiograph; ETOH, alcohol; GFR, glomerular filtration rate; Hb, haemoglobin; HDL, high density lipoprotein; HIV, Human Immunodeficiency Virus; HRT, hormone replacement therapy; HTN, hypertension; IHD, ischaemic heart disease; IS, ischaemic stroke; LAD, left anterior descending artery; LDL, low density lipoprotein; LVEF, left ventricular ejection fraction; LVH, left ventricular hypertrophy; MDRD, Modification of Diet in Renal Disease; MI, myocardial infarction; NIHSS, National Institute of Health Stroke Scale; NSAIDs, Nonsteroidal anti-inflammatory drugs; NYHA, New York Heart Association; PCI, percutaneous coronary intervention; PCR, protein:creatinine ratio; PP, pulse pressure; PVD, peripheral vascular disease; Rx, treatment; SBP, systolic blood pressure; SD, standard deviation; SES, socio-economic status; STS score, Society of Thoracic Surgery score; TAVI, transcatheter aortic valve implantation; TC, total cholesterol; TE, thromboembolic; TG, triglyceride; TIA, transient ischaemic attack.

TABLE OF CONTENTS

Page Number

Supplemental tables:

Appendix Table I. Search strategies……………………………………………………………………………3

Appendix Table II. Characteristics of included studies…………………………………………………………..4-18

Appendix Table III. Characteristics of studies included in the meta-analysis……………………………….19

Appendix Table IV: Quality assessment of studies using the Newcastle-Ottawa Scale for Cohort Studies……20

Appendix Table V. Subgroup analysis and meta-regression: the effect of study, participant and stroke………25-26 characteristics on the association between proteinuria and adjusted risk of stroke.

Appendix Table VI. Hierarchy of hypertension adjustment in studies included in the meta-analysis of………..30 proteinuria and stroke risk.

Supplemental figures:

Appendix Figure I: Unadjusted risk ratio (RR) for the association of proteinuria and stroke risk……………..21

Appendix Figure II: Risk ratio (RR) for the association of proteinuria and stroke risk using a fixed effects….22 model adjusted for traditional cardiovascular risk factors (exact methods varied between studies).

Appendix Figure III: Unadjusted (A) and adjusted (B) risk ratios (RR) for the association of………………...23 proteinuria and stroke risk using paired study estimates only.

Appendix Figure IV: Risk ratio (RR) for the association of proteinuria and (A) ischaemic stroke and (B)…....24 haemorrhagic stroke risk. RRs were adjusted for traditional cardiovascular risk factors (exact methods varied between studies).

Appendix Figure V: Impact of albuminuria level on stroke risk. (A) Studies reporting Microalbuminuria…….27 and (B) Macroalbuminuria.

Appendix Figure VI: Overall risk ratio (RR) for the association of combined albuminuria and low eGFR…….28 with stroke risk adjusted for traditional cardiovascular risk factors (exact methods varied between studies).

Appendix Figure VII: Funnel plot evaluating potential systematic bias in studies included in the……………...29 meta-analysis

Appendix Table I. Search strategies

MEDLINE	Embase
1. Kidney Diseases/<br>2. exp Renal Replacement Therapy/<br>3. Renal Insufficiency/<br>4. exp Renal Insufficiency, Chronic/<br>5. dialysis.tw.<br>6. (hemodialysis or haemodialysis).tw.<br>7. (hemofiltration or haemofiltration).tw.<br>8. (hemodiafiltration or haemodiafiltration).tw.<br>9. (end-stage renal or end-stage kidney or endstage renal or endstage kidney).tw.<br>10. (ESRF or ESKF or ESRD or ESKD).tw.<br>11. (chronic kidney or chronic renal).tw.<br>12. (CKF or CKD or CRF or CRD).tw.<br>13. (CAPD or CCPD or APD).tw.<br>14. (predialysis or pre-dialysis).tw.<br>15. or/1-14<br>16. exp Stroke/<br>17. Brain ischemia/<br>18. Cerebral Small Vessel Diseases/<br>19. Intracranial Hemorrhages/<br>20. stroke.tw.<br>21. (CVA or TIA).tw.<br>22. or/16-21<br>23. and/15,22<br>24. albuminuria/<br>25. proteinuria/<br>26. (proteinuria or albuminuria).tw.<br>27. or/24-26<br>28. or/15,27<br>29. and/22,28	1. kidney disease/<br>2. exp renal replacement therapy/<br>3. dialysis.tw.<br>4. (CKF or CKD or CRF or CRD).tw.<br>5. (end?stage kidney or end?stage renal).tw.<br>6. (chronic kidney or chronic renal).tw.<br>7. exp cerebrovascular accident/<br>8. brain hemorrhage/<br>9. brain infarction/<br>10. stroke.tw.<br>11. exp proteinuria/<br>12. albuminuria/<br>13. (proteinuria or ?albuminuria).tw.<br>14. or/1-6<br>15. or/7-10<br>16. or/11-13<br>17. or/14, 16<br>18. and/15, 17

Appendix Table II. Characteristics of included studies

**Study reference, Country, Name, (Reference)**	**Design, population, ethnicity**	**Size, (% men)**	**Mean or median age (SD or range)**	**GFR (ml/min/1.73m^2)**<br>**Formula**<br>***Reference*: range(n)**<br>***Comparison*: range (n)**	**Albuminuria (category)**<br>**Measurement**<br>***Reference*: range (n)**<br>***Comparison*: range (n)**	**Stroke type(n) (Classification)**	**Follow-up (months)**	**Other stroke characteristics**	**Adjustment (Hypertension)**
Aguilar 2010, USA, Cardio-vascular Health Study, (1)	Cohort,<br>No cerebrovascular disease,<br>17% Black.	3,205, (39)	78.5 (4.8)	MDRD<br>*Reference*:<br>≥60 (unknown)<br>*Comparison*:<br><60 (unknown)	ACR<br>*Reference*:<br>None (2,630)<br>*Comparison*:<br>Micro (560)<br>Macro (15)<br>Any (575)	Unspecified (26)<br>Ischaemic (316)<br>Haemorrhagic (48)	104.4	Incident<br>Fatal or non-fatal	Age, sex, race, BMI, smoking, hypertension, diabetes, LVH, AF, internal carotid artery stenosis ≥75%, SBP, DBP<br><br>(Categorical/Continuous variables - ≥140/90 or physician's diagnosis + use of Rx, SBP, DBP – average over 4-7 years)
Bello 2011, Canada, (2)	Cohort,<br>7% diabetics, 3% cerebrovascular disease,<br>22.3% HTN.<br>Unknown ethnicity.	1,023,686, (45)	48.7 (16.6)	MDRD<br>*Reference:*<br>≥60 (820,571)<br>*Comparison*:<br>45-60 (79, 845)<br>30-45 (16,713)<br>15-30 (3,856)	Dipstick, ACR<br>*Reference*:<br>None (913,830)<br>*Comparison*:<br>Micro (91,774)<br>Macro (18,082)	Unspecified (4,692)	35	Incident or recurrent<br>Fatal or non-fatal	Age, sex, diabetes, SES, previous malignancy, CVA, CCF, COPD, dementia, HIV, IHD, chronic liver disease, PVD, HTN.<br><br>(Categorical variable – history of HTN)
Da Costa 2017, Brazil, (3)	Cohort<br>100% resistant HTN.<br>47.5% DM, 24.2% CAD, 15.7% previous stroke, 9.7% smoking.	1048 (27.7)	70.6 (11.3)	CKD-Epi<br>*Reference:*<br>≥60 (637)<br>*Comparison:*<br>30-59 (363)<br><30 (48)	UAER<br>*Reference:*<br><30 (701)<br>*Comparison:*<br>≥30 (347)	Unspecified (90)	90	Incident<br>Fatal or non-fatal	Age, sex, DM, smoking, HDL-C, TG, LVMI, HF, PVD, uncontrolled ambulatory BP & non-dipping pattern.

									(Categorical variables: Uncontrolled ABPM = mean 24h >130/80, & non-dipping pattern)
De Leeuw 2002, Multinational, Syst-Eur trial, (4)	RCT, *Inclusion criteria*: isolated systolic hypertension, age≥60. *Intervention*: Ca-channel blocker +/- ACE *Control*: Placebo 11% diabetics, 30% previous cardiovascular disease, Unknown ethnicity.	4,658, (33)	70 (6.6)	Serum Creatinine Per 20 µmol/l increase	Dipstick *Reference*: None (4,225) *Comparison*: Micro (324) Macro (109)	Unspecified (129)	24	Incident Fatal or non-fatal	Active treatment, sex, age, systolic blood pressure smoking, previous cardiovascular disease, diabetes<br><br>(Continuous/categorical variables – on Rx, SBP – 6 readings in 1-month run-in)
Fuller 2001, Multinational, WHO multinational study of vascular disease in diabetes, (5)	Cohort, 100% diabetics, Unknown ethnicity	4,743, (49)	46.4 (5.8)		ACR *Reference*: None (unknown) *Comparison*: Micro (unknown); Macro (unknown)	Unspecified (293)	144	Incident or recurrent Fatal or non-fatal	Age, duration of DM, SBP, serum cholesterol, smoking status, proteinuria, retinopathy, & ECG abnormalities.<br><br>(Categorical/continuous variables - Hypertension = SBP >140 or DBP>90 or on treatment)
Go 2009, USA, (6)	Cohort, 17% diabetics, 9% cerebrovascular disease, 59% other vascular disease. 100% AF, 86% White, 4% black, 5% Asian.	13,535, (57)	71.6 (unknown)	MDRD *Reference*: ≥60 (13,535) *Comparison:* 45-60 (7,746) <45 (5,789)	Dipstick *Reference*: None (unknown) *Comparison*: Macro (unknown)	Ischaemic (637)	96	Incident or recurrent Fatal or non-fatal	Age, sex, race, SES, educational attainment, prior ischaemic stroke, CCF, diabetes, hypertension, IHD<br><br>(Categorical variable -

									Hypertension identified from outpatient sources.)
Hagg 2013,<br>Finland,<br>FinnDianne study, (7)	Cohort,<br>100% type 1 diabetics, 47% previous/current smokers, 7% dialysis-requiring ESKD.<br>Mainly white.	4,083, (52)	37.4 (11.8)		UAER<br>*Reference*:<br>None (2482)<br>*Comparison*:<br>Micro (510)<br>Macro (549)	Ischaemic (286)<br>Haemorrhagic (120).	108	Incident<br>Fatal or non-fatal	Age, sex, blood pressure, BMI, LDL/HDL cholesterol, TGs, smoking<br><br>(Continuous variable - Blood pressure was measured twice in the sitting position with a 10-min rest before the first measurement, and the mean values of these two measurements were calculated for both systolic blood pressure (SBP) and diastolic blood pressure (DBP).)
Hitman 2007,<br>UK,<br>CARDS trial, (8)	RCT,<br>*Inclusion criteria*: Type 2 diabetes and no previous cardiovascular disease<br>*Intervention*: Atorvastatin<br>*Control*: Placebo<br>0.4% AF, 22% smokers, 37% BMI>30, 79.4% HTN.	2838, (68)	62.1 (8.0)		Dipstick or ACR or AER<br>Reference:<br>None (2144)<br>Comparison:<br>Micro (694)	Unspecified (13)<br>Ischaemic (47)	46.8	Incident<br>Fatal or non-fatal	Age, sex, HbA1c>10%, treatment arm, SBP.<br><br>(Continuous variable)

Irie 2006, Japan, (9)	Cohort, 7% diabetics, Mainly Asian.	91,432, (34)	58.8 (unknown)	MDRD *Reference*: ≥100 (17,636) *Comparison*: 90-100(21,846) 80-90 (20,402) 70-80 (20,461) 60-70 (8,190) <60 (2,897)	Dipstick *Reference*: None (88,438) *Comparison*: Macro (1,929)	Unspecified (985)	120	Incident Fatal	Age, hypertension, smoking, ETOH, diabetes, total cholesterol, HDL cholesterol, BMI, urinary protein (for eGFR analyses) (Categorical variable – adjusted for hypertensive category).
Kowey 2005, Multinational, RENAAL study, (10)	RCT, *Inclusion criteria*: Type 2 diabetes with nephropathy *Intervention*: Losartan *Control*: Placebo 100% diabetics, 18% smoking. Unknown ethnicity.	1,513, (63)	60 (7.4)		ACR or 24 hour collection *Reference*: Micro (378) *Comparison*: Macro (1,135)	Unspecified (97)	40.8	Incident Fatal or non-fatal	Unadjusted
Lee 2016, South Korea, (11)	Cohort 74.9% HTN, 28.1% DM, 25.4% smoking, 19.7% IHD.	295 (53.2)	67.6 (14-94)	CKD-Epi *Reference:* ≥60 (239) *Comparison:* <60 (56)	UACR *Reference:* <30 (165) *Comparison:* ≥30 (130)	Ischemic (26) SAH (1)	22	Recurrent Fatal or non-fatal	Age, sex, DM, HTN, smoking, AF, previous stroke, alcohol Hx, NIHSS score. (Categorical variable – on treatment or SBP≥140 or DBP≥90 on repeated exam)
Li 2015, China, (12)	Cohort 43.6% HTN, 32.8% smoking, 9% DM.	92013 (70.6)	51.8	CKD-Epi *Reference:* ≥90 (30609) *Comparison:* 60-89 (49089) 30-60 (11801) <30 (514)	Urine dipstick *Reference:* None (88164) *Comparison:* ≥1+ (3849)	Ischaemic (1128) Haemorrhagic (406)	48	Incident	Age, sex, smoking, drinking, BMI, LDL-C, HDL-C, TG, TC, DM, HTN, hyperlipidaemia, AF. (Categorical variable - SBP≥140 or DBP≥90 or on Rx or self-reported Hx)

Madison 2006,<br>USA,<br>Honolulu heart study, (13)	Cohort,<br>15% diabetics, no known vascular disease, 51.6% HTN, 35.3% smokers.<br>100% Asian.	6,252,<br>(100)	60.0 (0.4)		Dipstick<br>*Reference*:<br>None (5802)<br>*Comparison*:<br>Macro (69)	Unspecified (457)	324	Incident<br>Fatal or non-fatal	Age, BMI, physical activity index, cholesterol, hypertension, diabetes, smoking, ETOH.<br><br>(Categorical variable - Hypertension was defined as systolic pressure of 140 mm Hg or higher or diastolic pressure of 90 mm Hg or higher, or there was documented use of antihypertensive agents.)
McAlister 2017, Canada, (14)	Cohort<br>100% AF, 64.1% HTN, 21.6% DM, 11.3% CAD.<br>Unknown ethnicity.	58451 (53.2)	66	CKD-Epi<br>*Reference:*<br>≥60 (44217)<br>*Comparison:*<br>45-59 (8046)<br>30-44 (4264)<br><30 (1924)	Urine dip/ACR/PCR<br>*Reference:*<br>Neg/<3/<15 (52132)<br>*Comparison:*<br>Trace or 1+/ 3-30/15-50 (3354)<br>2+/>30/>50 (2965)	Unspecified (5620)	31	Incident	Age, sex, aboriginal status, social assistance, postal code income quintile, rural/urban status, previous TE or bleeding event, CHF, HTN, DM, PVD.<br><br>(Categorical variable)

Menne 2014,<br>Multinational,<br>ROADMAP-OFU study,<br>(15)	Cohort<br>100% T2DM, 21.7% CAD, 15.8% smoking.<br>Unknown ethnicity.	1758 (48.9)	61.2 (8.4)		UACR<br>*Reference:*<br>None (1626)<br>*Comparison:*<br>30-300 (132)	Unspecified (26)	39.6	Incident<br>Fatal or non-fatal	Treatment strategy (olmesartan/placebo), SBP, DBP, HbA1c at baseline.<br><br>(Continuous variables)
Miettinen 1996,<br>Multinational,<br>(16)	Cohort,<br>43% diabetics,<br>Unknown ethnicity.	2,431,<br>(50)	58.1 (0.2)		PCR<br>*Reference*:<br>None (unknown)<br>*Comparison*:<br>Micro (unknown)<br>Overt proteinuria (unknown)	Unspecified (155)	84	Incident or recurrent<br>Fatal or non-fatal	Sex, age, location, previous stroke, TC, HDL-C, smoking, TG, HTN.<br><br>(Categorical variable – HTN = receiving drug treatment for hypertension or if systolic blood pressure was ≥160 mm Hg or diastolic blood pressure was ≥95 mm Hg measured in the sitting position after a 5-minute rest.)
Mikkelsen 2009,<br>Denmark, (17)	Cohort,<br>100% undergoing elective cardio-thoracic surgery, 15% DM, 57.1% HTN, 64.6% ever smoker, 15.1% previous AF.<br>Unknown ethnicity	962,<br>(73)	65.6<br>(18-93)		ACR<br>*Reference*:<br>None (782)<br>*Comparison*:<br>Micro (180)	Unspecified (38)	1	Incident or recurrent<br>Fatal or non-fatal	Unadjusted

Muntner 2012, USA , REGARDS study, (18)	Cohort, 13% smokers, 48% hypertensive, 37% black	20,386, (46)	64.4 (9.2)	CKD-EPI *Reference*: >90 (9,431) *Comparison*: 60-90 (9,053) 45-60 (1,321) <45 (581)	ACR *Reference*: None (13,310) *Comparison*: Micro (6,844) Macro (440)	Unspecified (2,548)	25.2	Incident Fatal or non-fatal	Age, race, sex, geographic region, education, household income, smoking, ETOH, BMI, systolic blood pressure, antihypertensive medication use, dyslipidemia, diabetes and CRP<br><br>(Continuous & Categorical variables – SBP, on Rx)
Nagai 2014, Japan, (19)	Cohort 26.3% HTN, 13.7% smoking.	298148 (39.7)	63.2 (8.1)	MDRD *Reference:* ≥60 (98987) *Comparison:* <60 (19391)	Urine dipstick *Reference:* Negative/trace (284567) *Comparison:* ≥1+ (13581)	Unspecified (4426)	36	Incident	Age, sex, BMI, HTN category, smoking, anti-dyslipidaemia drugs, hyperglycemia, hypoglycemic drugs.<br><br>(Categorical variable – HTN categories = normotensive, untreated, treated, drug-resistant. SBP≥140 or DBP≥90 mmHg)
Nakayama 1997, Japan, Shibata study, (20)	Cohort, 100% Asian.	2,302, (42)	Unknown		Dipstick *Reference*: Micro (unknown) *Comparison*: Macro (unknown)	Unspecified (28) Ischaemic (76) Haemorrhagic (38)	186	Incident Fatal or non-fatal	Age, blood pressure, Physical activity, Fundus abnormality, AF, Smoking, IHD<br><br>(Continuous variables - SBP, DBP, MBP)
Nakayama 2007, Japan, Okashama study, (21)	Cohort, Mainly Asian.	1,977, (37)	62.9	Cockcroft Gault *Reference*: >70 (555) *Comparison*: 40-70 (1,246)	Dipstick Reference: Micro (unknown) Comparison:	Unspecified (112)	96	Incident Fatal or non-fatal	Age, sex, systolic blood pressure, BMI, smoking, use of antihypertensive medication, history of

				<40 (176)	Macro<br>(unknown)				cardiovascular disease, hypercholesterolemia and diabetes<br><br>(Categorical and continuous variables – SBP, use of antihypertensive medications)
Oliveras 2003, Spain, (22)	Cohort,<br>100% renal transplant recipients.<br>6% diabetic, 13% IHD, 64.8% HTN, 17.1% smokers.<br>Unknown ethnicity.	403,<br>(37)	49.8<br>(23 to 63)		24 hour collection<br>*Reference*:<br><1g/24 hours (327)<br>*Comparison*:<br>>1g/24 hours (76)	Ischaemic (12)<br>Haemorrhagic (7)	120	Incident or recurrent<br>Fatal or non-fatal	(Categorical variable - Hypertension was defined either as a systolic blood pressure of 140 mmHg or higher and/or diastolic blood pressure of 90 mmHg or higher or cases in which treatment for HT had been implemented.)
Ravipati 2008, USA, (23)	Cohort,<br>100% diabetics or hypertensive.<br>54% White.	306,<br>(53)	57 (10)		ACR<br>*Reference*:<br>None (195)<br>*Comparison*:<br>Micro (111)	Unspecified (31)	39	Incident<br>Fatal or non-fatal	Unadjusted
Ruilope 2007, Multinational, VALUE trial, (24)	RCT,<br>*Inclusion criteria*:<br>Hypertensive, high cardiovascular risk.<br>*Intervention:* Valsartan<br>*Control:* Amlodipine,<br>89% white/4% black/4% Asian	15,245,<br>(58)	67.2 (8.1)	Cockcroft Gault<br>*Reference*:<br>>60 (9,214);<br>*Comparison*:<br><60 (5,999)	Dipstick<br>*Reference*:<br>None (11,788)<br>*Comparison*:<br>Any (3,435)	Unspecified (603)	45.6	Incident or recurrent<br>Fatal or non-fatal	Age, sex, IHD, LVH, all-cause death.<br><br>Not adjusted for hypertension.

Sander 2012, Germany, INSIGHT registry, (25)	Cohort,<br>35% diabetics, 79% HTN, 18% current smokers.	1,167 (58.1)	66 (11.9)		Dipstick<br>*Reference*:<br>None (781)<br>*Comparison*:<br>Micro (386)	Unspecified (35)	12	Recurrent<br>Fatal or non-fatal	Age, BMI, diabetes, SBP, total:HDL cholesterol, use of ACE/ARB, Ca-blockers, insulin, oral hypoglycemic, IHD, PAD, stroke subtype.<br><br>(Continuous variable – SBP)
Sandsmark 2015, USA, CRIC study, (26)	Cohort<br>55.2% DM, 33.9% CVD, 13.4% smoking. 42.4% White.	3939 (55.2)	58.1 (10.9)	MDRD<br>*Reference:*<br>>60 (702)<br>*Comparison:*<br>45-60 (1091)<br>30-44 (1339)<br><30 (807)	24h urine protein<br>*Reference:*<br><0.1g/24h (1375)<br>*Comparison:*<br>0.1-0.5 (1094)<br>0.5-1.5 (582)<br>>1.5 (690)	Unspecified (143)	76.8	Incident	Age, sex, race, DM, SBP, hyperlipidaemia, smoking, alcohol use.<br><br>(Continuous variable – baseline SBP, single reading)
Schrader 2006, Multinational, MARPLE study, (27)	Cohort study,<br>100% hypertensive,<br>Unknown ethnicity.	3,529, (43)	63 (8.3)		ACR<br>*Reference*:<br>None (1,750)<br>*Comparison*:<br>Micro (832)<br>Overt proteinuria (118)	Unspecified (24)	42.5	Incident or recurrent<br>Non-fatal	Unadjusted
Tanaka 1985, Japan, (28)	Cohort,<br>Unknown ethnicity.	2,299, (42)	All ≥ 40		Dipstick<br>*Reference*:<br><Macro (2,143)<br>*Comparison*:<br>Macro (156)	Ischaemic (34)	20	Incident	Age<br><br>(Continuous variables – SBP, DBP, MBP)
Tebbe 2010, Germany, (29)	Cohort<br>22.6% DM, 12.1% current smoker, 6.8% AF,<br>Unknown ethnicity.	2,173, (48)	61.4 (11.3)		ACR<br>*Reference*:<br>None (1,382)<br>*Comparison*:<br>Micro (791)	Unspecified (5)	12	Incident or recurrent<br>Non-fatal	Age<br><br>Not adjusted for hypertension.

Tonelli 2005, USA, CARE trial, (30)	RCT, *Inclusion criteria:* Hyperlipidemia and previous MI *Intervention:* Pravastatin *Control:* Placebo, Unknown ethnicity.	4,098, (86)	59.7 (50-70)	MDRD *Reference*: >60 (3,218) *Comparison*: <60 (880)	Dipstick *Reference*: None (3,546) *Comparison*: Macro (552)	Unspecified (130)	58.9	Non-fatal	Age, sex, ethnic origin, smoking, BMI, waist: hip ratio, fasting glucose, hemoglobin, albumin, LDL/HDL cholesterol, TGs, systolic/diastolic blood pressure, location, LVEF, use of drugs (ACEi, aspirin, or pravastatin).<br><br>(Continuous variables – SBP/DBP)
Valmadrid 2000, USA, (31)	Cohort, 100% diabetics, 65.9% HTN, 47.2% ever smoker. Unknown ethnicity.	840, (45)	67.9 (11.0)		Agglutination assay *Reference*: None (460) *Comparison*: Micro (208) Overt proteinuria (172)	Unspecified (85)	144	Incident or recurrent Fatal or non-fatal	Age, sex, glycemic control, insulin use, ETOH use, physical activity, history of cardiovascular disease, use of antihypertensive medication, severity of diabetic retinopathy.<br><br>history of hypertension (defined as systolic blood pressure of ≥160 mm Hg or a diastolic of ≥95 mm Hg or taking antihypertensive medications).<br><br>(Categorical variable – hypertension history or on meds)

Vernooij 2013, Netherlands, SMART study, (32)	Cohort<br>94.3% HTN, 65.3% IHD, 36% smoking, 18.3% T2DM.<br>Unknown ethnicity.	4319 (79.6)	65.5 (10.8)	MDRD<br>*Reference:*<br>≥60 (3672)<br>*Comparison:*<br><60 (647)	UACR<br>*Reference:*<br>≤3.0 mg/mmol (3558)<br>*Comparison:*<br>>3.0 (761)	Unspecified (156)	52.8	Incident	Age, sex, BMI, SBP, antihypertensive Rx, T2DM, CAD.<br><br>(Categorical & continuous variables – on Rx, SBP)
Wagener 1994, USA, NHANES (1/2/3), (33)	Cohort,<br>13% IHD, 30% smokers, 23% HTN, 7.1% DM.<br>100% White.	6,135, (47)	45-74		Dipstick<br>*Reference*:<br>None (5,952)<br>*Comparison*:<br>Any (183)	Unspecified (771)	192	Incident or recurrent<br>Fatal or non-fatal	Age, systolic blood pressure, diabetes, IHD, education, smoking<br><br>(Continuous variable – SBP)
Yang 2008, Hong Kong, (34)	Cohort,<br>100% T2DM, 19.4% current smokers.<br>Mainly Asian.	6,969, (46)	57<br>(46-67)	MDRD<br>*Reference:*<br>>115 (2,622)<br>*Comparison*:<br>60-115 (3,704)<br><60 (643)	ACR<br>*Reference*:<br>None (4,008)<br>*Comparison*:<br>Any (2961)	Ischaemic (314)	64.3	Incident<br>Fatal or non-fatal	Age, sex, BMI, smoking, hyperlipidemia, antihypertensive medication, SBP/DBP<br><br>(Continuous variables – SBP/DBP)
Yokota 2008, Japan, (35)	Cohort,<br>100% admitted with stroke, 84% HTN, 31% diabetics, 26% AF, 15% IHD.<br>Unknown ethnicity	474, (66)	70 (11)		ACR<br>*Reference:*<br>None (309)<br>*Comparison:*<br>Micro (133)<br>Macro (32)	Ischaemic (49)<br>Haemorrhagic (5)	12.8	Recurrent<br>Fatal or non-fatal	Sex, diabetes<br><br>(Not adjusted for HTN)
Yuyun 2004, UK, EPIC-Norfolk study, (36)	Cohort,<br>2% diabetics, 41% smokers, 13% HTN, no previous cerebrovascular disease.	23,630, (46)	59.0 (9.3)		ACR<br>*Reference*:<br>None (20,684)<br>*Comparison*:<br>Micro (2,749)<br>Macro (197)	Unspecified (85)<br>Ischaemic (112)<br>Haemorrhagic (49)	86.4	Incident<br>Fatal or non-fatal	Age, sex, smoking, use of antihypertensive medication, systolic blood pressure, total cholesterol, diabetes, BMI, family history of stroke, and baseline coronary heart disease

									(Categorical variable [hypertension] and continuous variable [sbp – per SD])
Zhang 2008, USA, (37)	Cohort,<br>48.8% DM, 39.2% HTN. 33.9% current smokers.<br>100% American-Indian.	4,549, (40)	56.3 (8.0)		ACR<br>*Reference*:<br>None (3,084)<br>*Comparison*:<br>Micro (831)<br>Macro (464)	Unspecified (48)<br>Ischaemic (221)<br>Haemorrhagic (37)	160.8	Incident<br>Fatal or non-fatal	Age, sex, blood pressure, BMI, waist circumference, LDL/HDL cholesterol, TGs, physical activity, fasting glucose, smoking, ETOH<br><br>Hypertension was defined by the criteria of the seventh report of the Joint National Committee on Prevention, Detection, Evaluation, and Treatment of Hypertension (JNC-7; systolic blood pressure ≥140 mm Hg, diastolic blood pressure ≥90 mm Hg, or use of antihypertensive medication). Prehypertension was defined as systolic blood pressure 120 to 139 mm Hg or diastolic blood pressure 80 to 89 mm Hg. Normal blood pressure was defined as <120/80 mm Hg.

									(Categorical [preHTN & HTN] and continuous variables [SBP & DBP})
Zhang 2015, China, CSPPT, (38)	RCT<br>*Inclusion criteria:*<br>45-75 yrs with HTN<br>*Intervention:*<br>Enalapril & folic acid<br>*Control:*<br>31% smoking, 11.1% DM.	19599 (40.8)	60 (7.5)	CKD-Epi<br>*Reference:*<br>≥90 (13418)<br>*Comparison:*<br>60-89 (5768)<br><60 (413)	Urine dipstick<br>*Reference:*<br>None (16663)<br>*Comparison:*<br>Trace (1812)<br>≥1+ (1154)	Ischaemic (472)<br>Haemorrhagic (111)<br>Undefined (2)	54	Incident	Age, study center, gender, treatment group, smoking, alcohol, BMI, baseline SBP/DBP, mean SBP/DBP over treatment period, TC, HDL, FPG, homocysteine, folate.<br><br>(Continuous variables – baseline & mean)

Abbreviations:

AAA, abdominal aortic aneurysm; ACE, Angiotensin Converting Enzyme inhibitor; ACR, albumin:creatinine ratio; AF, atrial fibrillation; AR, aortic regurgitation; A2RB, Angiotensin 2 Receptor Blocker; BMI, body mass index; BMS, bare metal stent; Ca, calcium; CABG, coronary artery bypass grafting; CAD, coronary artery disease; CCF, congestive cardiac failure; CEA, carotid endarterectomy; CLD, chronic liver disease; COPD, chronic obstructive pulmonary disease; CRP, C Reactive Protein; CVA, cerebrovascular accident; CVD, cardiovascular disease; DAPT, dual anti-platelet therapy; DBP, diastolic blood pressure; DM, diabetes mellitus; ECG, electrocardiograph; ETOH, alcohol; GFR, glomerular filtration rate; Hb, haemoglobin; HDL, high density lipoprotein; HIV, Human Immunodeficiency Virus; HRT, hormone replacement therapy; HTN, hypertension; IHD, ischaemic heart disease; IS, ischaemic stroke; LAD, left anterior descending artery; LDL, low density lipoprotein; LVEF, left ventricular ejection fraction; LVH, left ventricular hypertrophy; MDRD, Modification of Diet in Renal Disease; MI, myocardial infarction; NIHSS, National Institute of Health Stroke Scale; NSAIDs, Nonsteroidal anti-inflammatory drugs; NYHA, New York Heart Association; PCI, percutaneous coronary intervention; PCR, protein:creatinine ratio; PP, pulse pressure; PVD, peripheral vascular disease; Rx, treatment; SBP, systolic blood pressure; SD, standard deviation; SES, socio-economic status; STS score, Society of Thoracic Surgery score; TAVI, transcatheter aortic valve implantation; TC, total cholesterol; TE, thromboembolic; TG, triglyceride; TIA, transient ischaemic attack.

References to included studies:

1. Aguilar MI, O'Meara ES, Seliger S, Longstreth WT, Jr., Hart RG, Pergola PE, et al. Albuminuria and the risk of incident stroke and stroke types in older adults. Neurology. 2010;75(15):1343-50.
2. Bello AK, Hemmelgarn B, Lloyd A, James MT, Manns BJ, Klarenbach S, et al. Associations among estimated glomerular filtration rate, proteinuria, and adverse cardiovascular outcomes. Clin J Am Soc Nephrol. 2011;6(6):1418-26.
3. da Costa PM, Cortez AF, de Souza F, Mares GDS, Dos Santos BDM, Muxfeldt ES. Prognostic impact of baseline urinary albumin excretion rate in patients with resistant hypertension: a prospective cohort study. J Hum Hypertens. 2017:1-11.
4. De Leeuw PW, Thijs L, Birkenhager WH, Voyaki SM, Efstratopoulos AD, Fagard RH, et al. Prognostic significance of renal function in elderly patients with isolated systolic hypertension: results from the Syst-Eur trial. J Am Soc Nephrol. 2002;13(9):2213-22.
5. Fuller JH, Stevens LK, Wang SL. Risk factors for cardiovascular mortality and morbidity: the WHO Mutinational Study of Vascular Disease in Diabetes. Diabetologia. 2001;44 Suppl 2:S54-64.
6. Go AS, Fang MC, Udaltsova N, Chang Y, Pomernacki NK, Borowsky L, et al. Impact of proteinuria and glomerular filtration rate on risk of thromboembolism in atrial fibrillation: the anticoagulation and risk factors in atrial fibrillation (ATRIA) study. Circulation. 2009;119(10):1363-9.
7. Hagg S, Thorn LM, Putaala J, Liebkind R, Harjutsalo V, Forsblom CM, et al. Incidence of stroke according to presence of diabetic nephropathy and severe diabetic retinopathy in patients with type 1 diabetes. Diabetes Care. 2013;36 (12):4140-6.
8. Hitman GA, Colhoun H, Newman C, Szarek M, Betteridge DJ, Durrington PN, et al. Stroke prediction and stroke prevention with atorvastatin in the Collaborative Atorvastatin Diabetes Study (CARDS). Diabet Med. 2007;24 (12):1313-21.
9. Irie F, Iso H, Sairenchi T, Fukasawa N, Yamagishi K, Ikehara S, et al. The relationships of proteinuria, serum creatinine, glomerular filtration rate with cardiovascular disease mortality in Japanese general population. Kidney Int. 2006;69(7):1264-71.
10. Kowey PR, Dickson TZ, Zhang Z, Shahinfar S, Brenner BM, Investigators R. Losartan and end-organ protection--lessons from the RENAAL study. Clin Cardiol. 2005;28(3):136-42.
11. Lee SJ, Lee DG. Relationship between Kidney Dysfunction and Ischemic Stroke Outcomes: Albuminuria, but Not Estimated Glomerular Filtration Rate, Is Associated with the Risk of Further Vascular Events and Mortality after Stroke. PLoS ONE. 2016;11(5):e0155939.
12. Li Z, Wang A, Cai J, Gao X, Zhou Y, Luo Y, et al. Impact of proteinuria and glomerular filtration rate on risk of ischaemic and intracerebral hemorrhagic stroke: A result from the Kailuan study. Eur J Neurol. 2015;22(2):355-60.
13. Madison JR, Spies C, Schatz IJ, Masaki K, Chen R, Yano K, et al. Proteinuria and risk for stroke and coronary heart disease during 27 years of follow-up: the Honolulu Heart Program. Arch Intern Med. 2006;166(8):884-9.
14. McAlister FA, Wiebe N, Jun M, Sandhu R, James MT, McMurtry MS, et al. Are Existing Risk Scores for Nonvalvular Atrial Fibrillation Useful for Prediction or Risk Adjustment in Patients With Chronic Kidney Disease? Can J Cardiol. 2017;33(2):243-52.
15. Menne J, Ritz E, Ruilope LM, Chatzikyrkou C, Viberti G, Haller H. The randomized olmesartan and diabetes microalbuminuria prevention (ROADMAP) observational follow-up study: Benefits of RAS blockade with olmesartan treatment are sustained after study discontinuation. J Am Heart Assoc. 2014;3 (2):e000810.
16. Miettinen H, Haffner SM, Lehto S, Ronnemaa T, Pyorala K, Laakso M. Proteinuria predicts stroke and other atherosclerotic vascular disease events in nondiabetic and non-insulin-dependent diabetic subjects. Stroke. 1996;27(11):2033-9.
17. Mikkelsen MM, Andersen NH, Christensen TD, Hansen TK, Eiskjaer H, Mogensen CE, et al. Microalbuminuria and short-term prognosis in patients undergoing cardiac surgery. Interact Cardiovasc Thorac Surg. 2009;9(3):484-90.
18. Muntner P, Judd SE, McClellan W, Meschia JF, Warnock DG, Howard VJ. Incidence of stroke symptoms among adults with chronic kidney disease: results from the REasons for Geographic And Racial Differences in Stroke (REGARDS) study. Nephrol Dial Transplant. 2012;27(1):166-73.
19. Nagai K, Yamagata K, Ohkubo R, Saito C, Asahi K, Iseki K, et al. Annual decline in estimated glomerular filtration rate is a risk factor for cardiovascular events independent of proteinuria. Nephrology. 2014;19(9):574-80.
20. Nakayama T, Date C, Yokoyama T, Yoshiike N, Yamaguchi M, Tanaka H. A 15.5-year follow-up study of stroke in a Japanese provincial city. The Shibata Study. Stroke. 1997;28(1):45-52.

21. Nakayama M, Metoki H, Terawaki H, Ohkubo T, Kikuya M, Sato T, et al. Kidney dysfunction as a risk factor for first symptomatic stroke events in a general Japanese population--the Ohasama study. Nephrol Dial Transplant. 2007;22(7):1910-5.
22. Oliveras A, Roquer J, Puig JM, Rodriguez A, Mir M, Orfila MA, et al. Stroke in renal transplant recipients: epidemiology, predictive risk factors and outcome. Clin Transplant. 2003;17(1):1-8.
23. Ravipati G, Aronow WS, Ahn C, Alappat RM, McClung JA, Weiss MB. Incidence of new stroke or new myocardial infarction or death at 39-month follow-up in patients with diabetes mellitus, hypertension or both with and without microalbuminuria. Cardiology. 2008;109(1):62-5.
24. Ruilope LM, Zanchetti A, Julius S, McInnes GT, Segura J, Stolt P, et al. Prediction of cardiovascular outcome by estimated glomerular filtration rate and estimated creatinine clearance in the high-risk hypertension population of the VALUE trial. J Hypertens. 2007;25(7):1473-9.
25. Sander D, Weimar C, Bramlage P, Brandt T, Rosin L, Siebler M. Microalbuminuria indicates long-term vascular risk in patients after acute stroke undergoing in-patient rehabilitation. BMC Neurol. 2012;12(102).
26. Sandsmark DK, Messe SR, Zhang X, Roy J, Nessel L, Lee Hamm L, et al. Proteinuria, but Not eGFR, Predicts Stroke Risk in Chronic Kidney Disease: Chronic Renal Insufficiency Cohort Study. Stroke. 2015;46(8):2075-80.
27. Schrader J, Luders S, Kulschewski A, Hammersen F, Zuchner C, Venneklaas U, et al. Microalbuminuria and tubular proteinuria as risk predictors of cardiovascular morbidity and mortality in essential hypertension: final results of a prospective long-term study (MARPLE Study). J Hypertens. 2006;24(3):541-8.
28. Tanaka H, Hayashi M, Date C, Imai K, Asada M, Shoji H, et al. Epidemiologic studies of stroke in Shibata, a Japanese provincial city: preliminary report on risk factors for cerebral infarction. Stroke. 1985;16(5):773-80.
29. Tebbe U, Bramlage P, Luders S, Cuneo A, Sistig P, de Haan F, et al. Follow-up of cardiovascular risk markers in hypertensive patients treated with irbesartan: results of the i-SEARCH Plus Registry. J Clin Hypertens. 2010;12(12):909-16.
30. Tonelli M, Jose P, Curhan G, Sacks F, Braunwald E, Pfeffer M, et al. Proteinuria, impaired kidney function, and adverse outcomes in people with coronary disease: analysis of a previously conducted randomised trial. BMJ. 2006;332(7555):1426.
31. Valmadrid CT, Klein R, Moss SE, Klein BE. The risk of cardiovascular disease mortality associated with microalbuminuria and gross proteinuria in persons with older-onset diabetes mellitus. Arch Intern Med. 2000;160(8):1093-100.
32. Vernooij JWP, Van Der Graaf Y, Nathoe HM, Bemelmans RHH, Visseren FLJ, Spiering W. Hypertensive target organ damage and the risk for vascular events and all-causemortality in patients with vascular disease. J Hypertens. 2013;31(3):492-500.
33. Wagener DK, Harris T, Madans JH. Proteinuria as a biomarker: risk of subsequent morbidity and mortality. Environ Res. 1994;66(2):160-72.
34. Yang X, So W-Y, Ma RC, Ko GT, Kong AP, Ho C-S, et al. Thresholds of risk factors for ischemic stroke in type 2 diabetic patients with and without albuminuria: a non-linear approach. Clin Neurol Neurosurg. 2008;110(7):701-9.
35. Yokota C, Minematsu K, Ito A, Toyoda K, Nagasawa H, Yamaguchi T. Albuminuria, but not metabolic syndrome, is a significant predictor of stroke recurrence in ischemic stroke. J Neurol Sci. 2009;277 (1-2):50-3.
36. Yuyun MF, Khaw KT, Luben R, Welch A, Bingham S, Day NE, et al. Microalbuminuria and stroke in a British population: the European Prospective Investigation into Cancer in Norfolk (EPIC-Norfolk) population study. J Intern Med. 2004;255(2):247-56.
37. Zhang Y, Galloway JM, Welty TK, Wiebers DO, Whisnant JP, Devereux RB, et al. Incidence and risk factors for stroke in American Indians: the Strong Heart Study. Circulation. 2008;118(15):1577-84.
38. Zhang C, Wang X, He M, Qin X, Tang G, Wang Y, et al. Proteinuria is an independent risk factor for first incident stroke in adults under treatment for hypertension in China. J Am Heart Assoc. 2015;4 (12):e002639.

Appendix Table III. Characteristics of studies included in the meta-analysis

Characteristics	Number of studies, total = 38<br>N	 %
**Study**		
**Design**		
Randomized controlled trial	6	15.8
Cohort study	32	84.2
**Location**		
North America	12	31.6
South America	1	2.6
Europe	8	21.1
Asia	10	26.3
Multinational	7	18.4
**Number of participants**		
0 to <2500	14	36.8
≥2500 to <5000	11	28.9
≥5000 to <20000	5	13.2
≥20000	8	21.1
**Duration of follow-up (months)**		
0 to <24	5	13.2
≥24 to <60	16	42.1
≥60 to <96	5	13.2
≥96	12	31.6
**Decade of publication**		
1980s	1	2.6
1990s	3	7.9
2000s	19	50.0
2010s	15	39.5
**Participant**		
**Mean age (years)**		
<60	13	34.2
≥60 to <65	10	26.3
≥65 to <70	7	18.4
≥70	5	13.2
**Hypertensives (%)**		
<25	4	10.5
≥25 to <50	5	13.2
≥50 to <75	7	18.4
≥75	12	31.6
**Diabetics (%)**		
<15	11	28.9
≥15 to <30	10	26.3
≥30	14	36.8
**Stroke**		
**Subtype**		
Unspecified	30	78.9
Ischaemic	14	36.8
Haemorrhagic	10	26.3

Appendix Table IV: Quality assessment of studies using the Newcastle-Ottawa Scale for Cohort Studies

Good quality	Fair quality	Poor quality
Aguilar 2010<br>Bello 2011<br>Da Costat 2017<br>De Leeuw 2002<br>Fuller 2001<br>Go 2009<br>Hagg 2013<br>Hitman 2007<br>Irie 2006<br>Kowey 2005<br>Lee 2016<br>Li 2015<br>Madison 2006<br>McAlister 2017<br>Menne 2014<br>Miettinen 1996<br>Mikkelsen 2009<br>Muntner 2012<br>Nagai 2014<br>Nakayama 1997<br>Nakayama 2007<br>Ruilope 2007<br>Sander 2012<br>Sandsmark 2015<br>Schrader 2006<br>Tebbe 2010<br>Tonelli 2006<br>Valmadrid 2000<br>Vernooij 2013<br>Wagner 1994<br>Yang 2008<br>Yokoto 2008<br>Yuyun 2004<br>Zhang 2008<br>Zhang 2015	Tanaka 1985 *(Analysis not controlled for all confounders and no confidence intervals reported for effect estimates)*	Oliveras 2003 *(Selected group, retrospective, ascertainment method of exposure unclear, unadjusted analysis, assessment of outcome was not record linked or unblinded, loss to follow-up)*<br>Ravipati 2008 *(Unclear adjustment for confounders for the microalbuminuria analysis with no adjusted effect estimates provided. No statement about assessment of outcome)*

Thresholds for converting the Newcastle-Ottawa scales to AHRQ standards (good, fair, and poor):
Good quality: 3 or 4 stars in selection domain AND 1 or 2 stars in comparability domain AND 2 or 3 stars in outcome/exposure domain
Fair quality: 2 stars in selection domain AND 1 or 2 stars in comparability domain AND 2 or 3 stars in outcome/exposure domain
Poor quality: 0 or 1 star in selection domain OR 0 stars in comparability domain OR 0 or 1 stars in outcome/exposure domain

Appendix Figure I: Unadjusted risk ratio (RR) for the association of proteinuria and stroke risk.

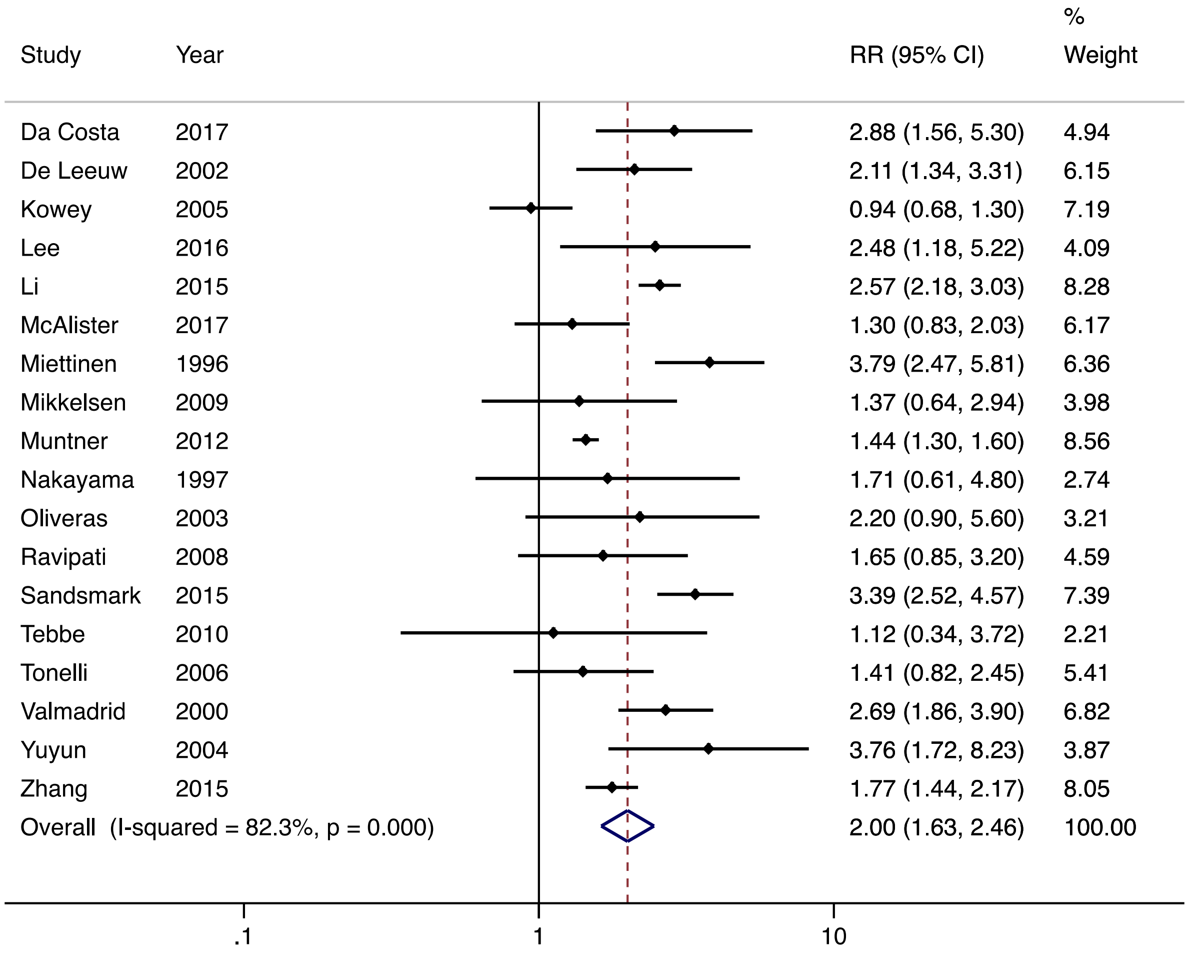

Appendix Figure II: Risk ratio (RR) for the association of proteinuria and stroke risk using a fixed effects model adjusted for traditional cardiovascular risk factors (exact methods varied between studies).

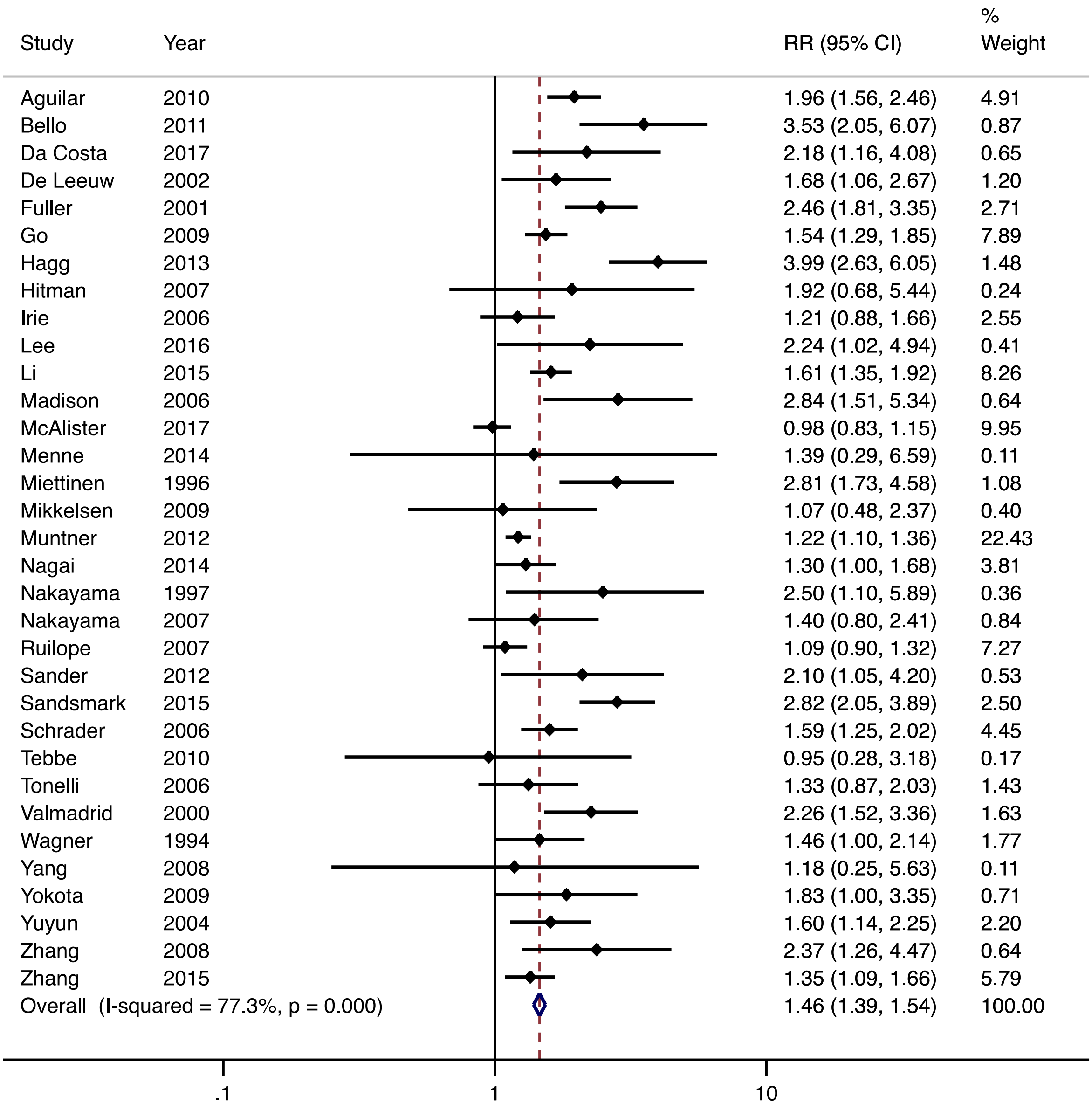

Appendix Figure III: Unadjusted (A) and adjusted (B) risk ratios (RR) for the association of proteinuria and stroke risk using paired study estimates only.

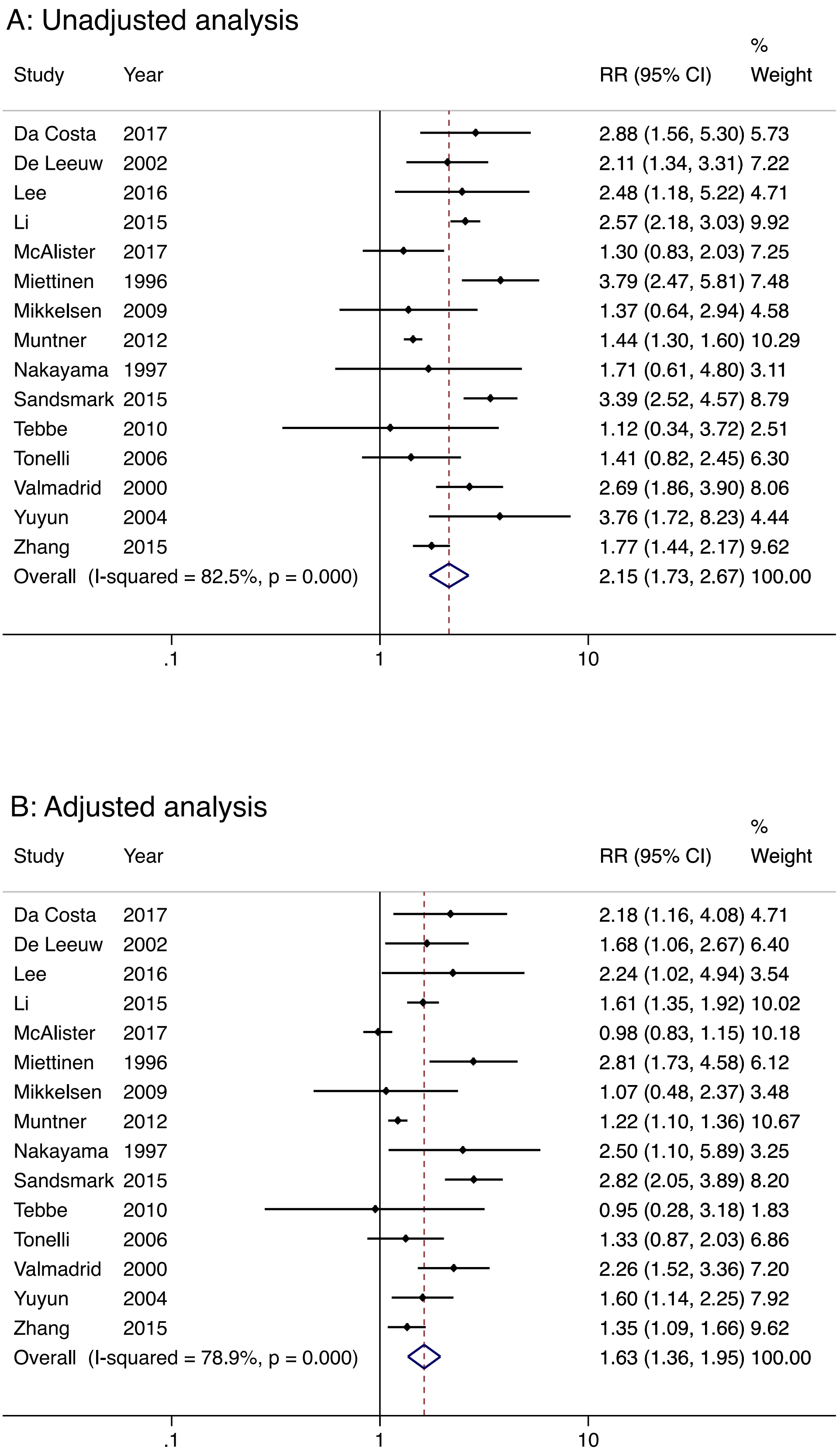

Appendix Figure IV: Risk ratio (RR) for the association of proteinuria and (A) ischaemic stroke and (B) haemorrhagic stroke risk. RRs were adjusted for traditional cardiovascular risk factors (exact methods varied between studies).

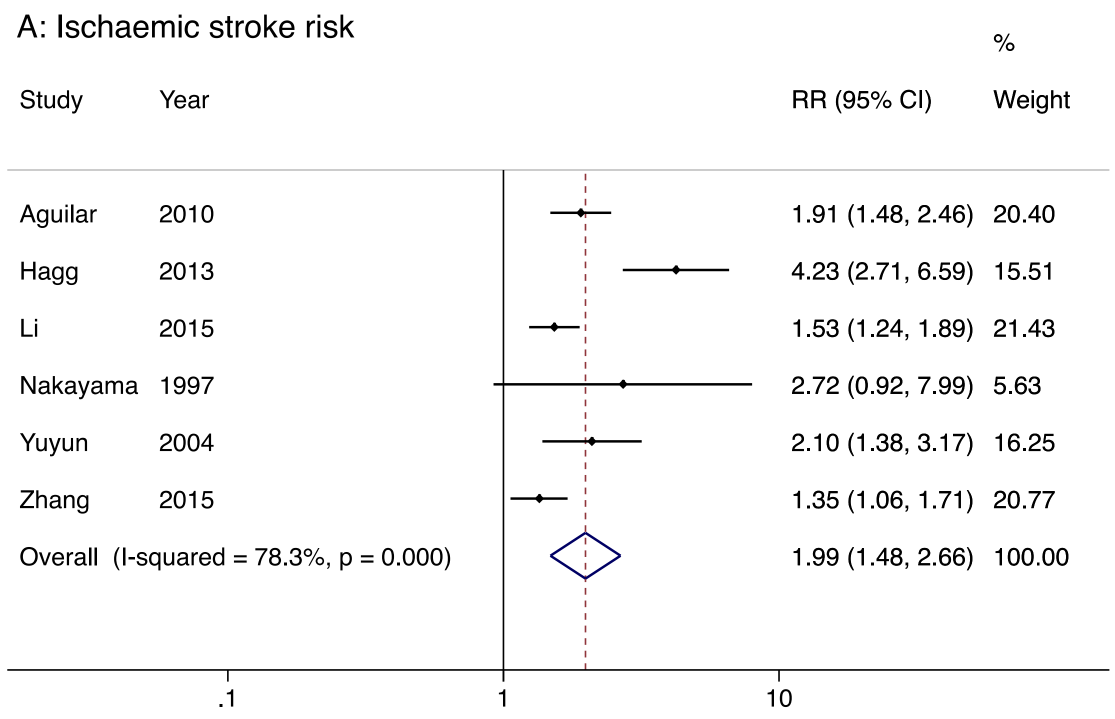

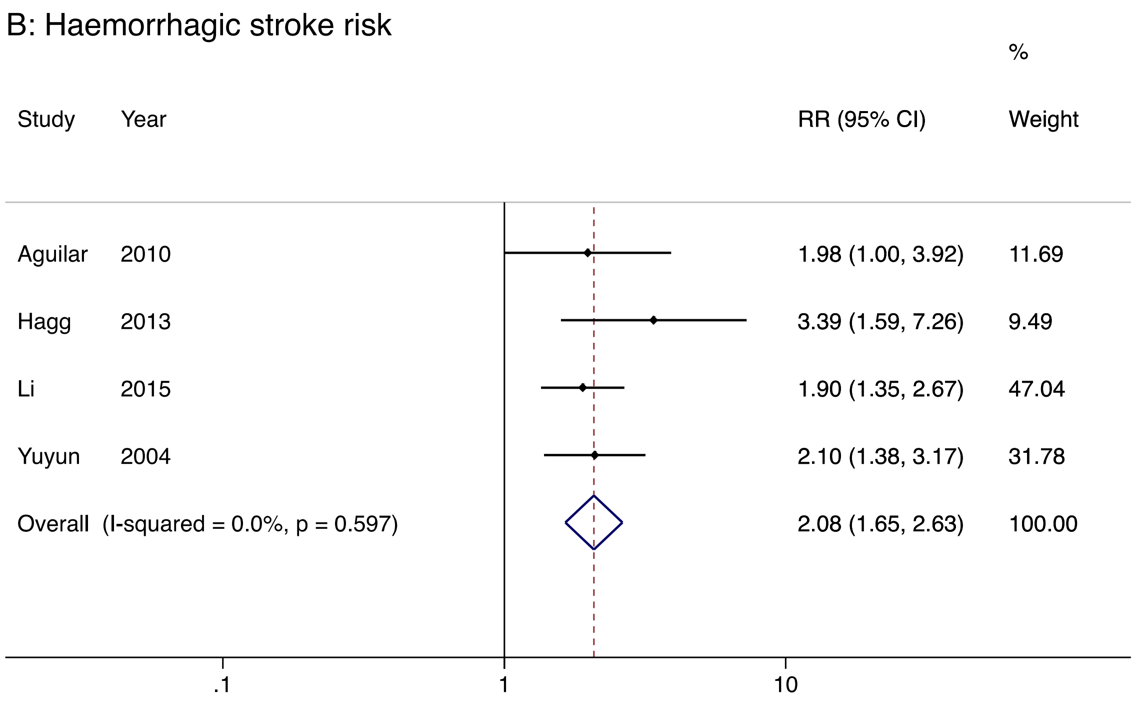

Appendix Table V. Subgroup analysis and meta-regression: the effect of study, participant and stroke characteristics on the association between proteinuria and adjusted risk of stroke.

**Subgroups**	**Number of studies**	**RR (95% CI)**	**P value for heterogeneity**
**Study characteristics**			
Design			
Cohort	28	1.80 (1.56-2.08)	0.12
Randomized controlled trial	5	1.27 (1.09-1.48)	
Location			
North America	11	1.78 (1.42-2.22)	0.4
Europe	6	1.88 (1.18-3.00)	
Asia	9	1.45 (1.31-1.62)	
Multinational	6	1.75 (1.25-2.47)	
South America	1	2.18 (1.16-4.09)	
Size			
0 to <5000	20	2.05 (1.78 to 2.37)	0.002
≥20000	13	1.40 (1.22 to 1.60)	
Duration of follow-up (months)			
0 to <24	4	1.63 (1.08-2.46)	0.39
≥24 to <60	13	1.39 (1.21-1.60)	
≥60 to <96	5	2.23 (1.66-2.99)	
≥96	11	1.98 (1.61-2.44)	
Albuminuria quantification			
Urine dipstick	13	1.43 (1.28-1.60)	0.06
Laboratory methods	20	1.94 (1.57-2.39)	
**Patient characteristics**			
Mean age (years)			
<60	11	2.12 (1.65-2.70)	0.02
≥60	22	1.50 (1.32-1.70)	

Gender			
Mainly male	14	1.80 (1.42-2.28)	0.7
Mainly female	19	1.66 (1.44-1.92)	
Race			
Mainly Caucasian	10	1.60 (1.31-1.94)	0.71
Mainly Asian	10	1.49 (1.31-1.70)	
Diabetics (%)			
<15	10	1.59 (1.40-1.82)	0.08
≥15 to <30	9	1.38 (1.13-1.69)	
≥30	12	2.18 (1.60-2.99)	
Hypertensives (%)			
<25	4	1.80 (1.25-2.58)	0.74
≥25 to <50	5	1.41 (1.18-1.67)	
≥50 to <75	6	1.63 (1.17-2.26)	
≥75	10	1.49 (1.26-1.76)	
Smokers (%)			
<15	6	1.64 (1.18-1.26)	0.38
≥15 to <20	5	1.46 (1.09-1.95)	
≥20 to <30	3	1.44 (0.86-2.40)	
≥30	10	1.88 (1.54-2.29)	
Atrial fibrillation (%)			
<10	5	1.83 (1.48-2.26)	0.16
≥10	4	1.29 (0.92-1.82)	
Stroke type			
Incident	20	1.67 (1.42-1.96)	0.23
Recurrent	2	2.16 (1.28-3.64)	
Incident or recurrent	10	1.77 (1.40-2.23)	

GFR; glomerular filtration rate, RR; relative risk, CI; confidence interval
MDRD; modification of diet in renal disease, CKD-EPI; chronic kidney disease epidemiology collaboration
UACR; urine albumin-creatinine ratio, UAER; urine albumin excretion rate, UPCR; urine protein-creatinine ratio

Appendix Figure V: Impact of albuminuria level on stroke risk. (A) Studies reporting Microalbuminuria and (B) Macroalbuminuria.

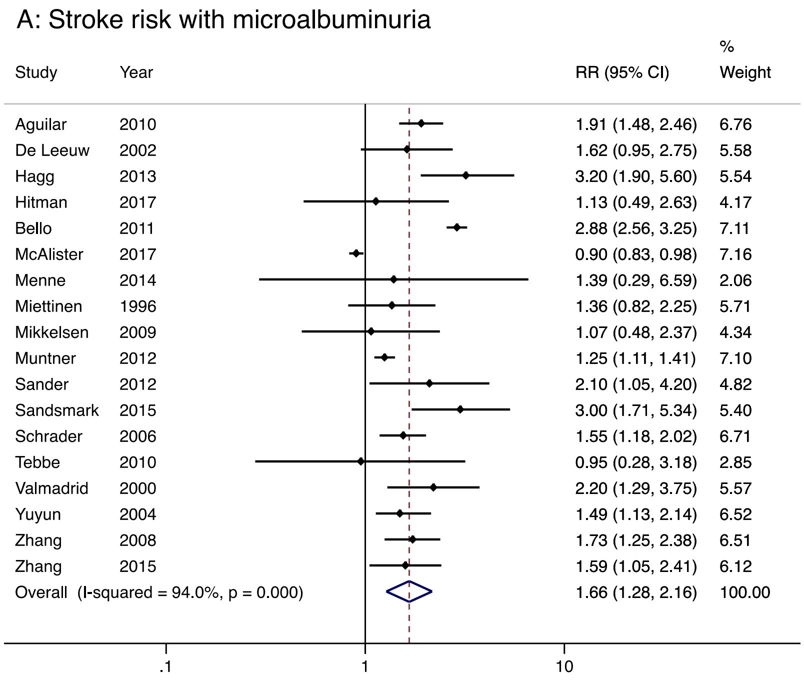

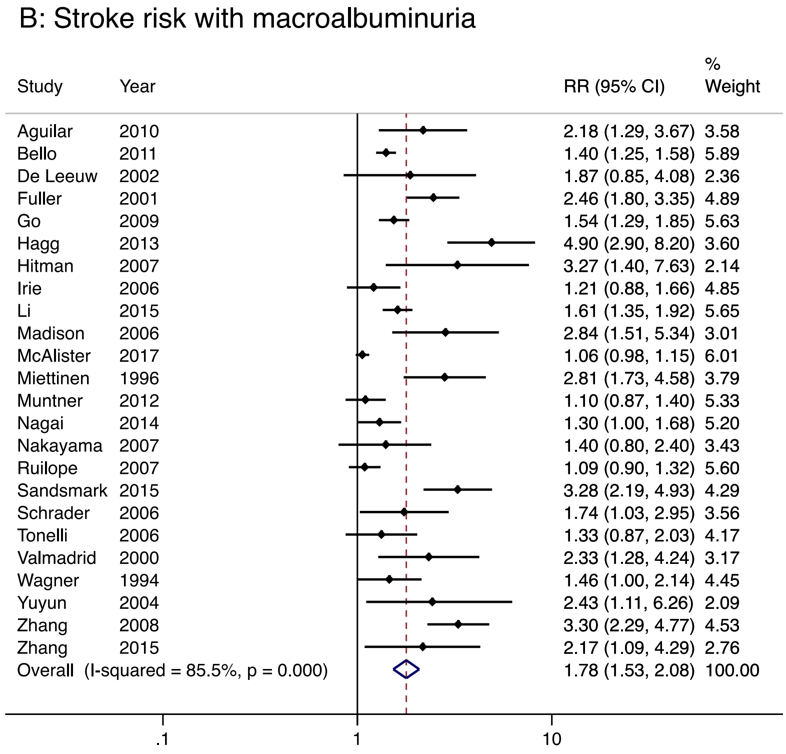

Appendix Figure VI: Overall risk ratio (RR) for the association of combined albuminuria and low eGFR with stroke risk adjusted for traditional cardiovascular risk factors (exact methods varied between studies).

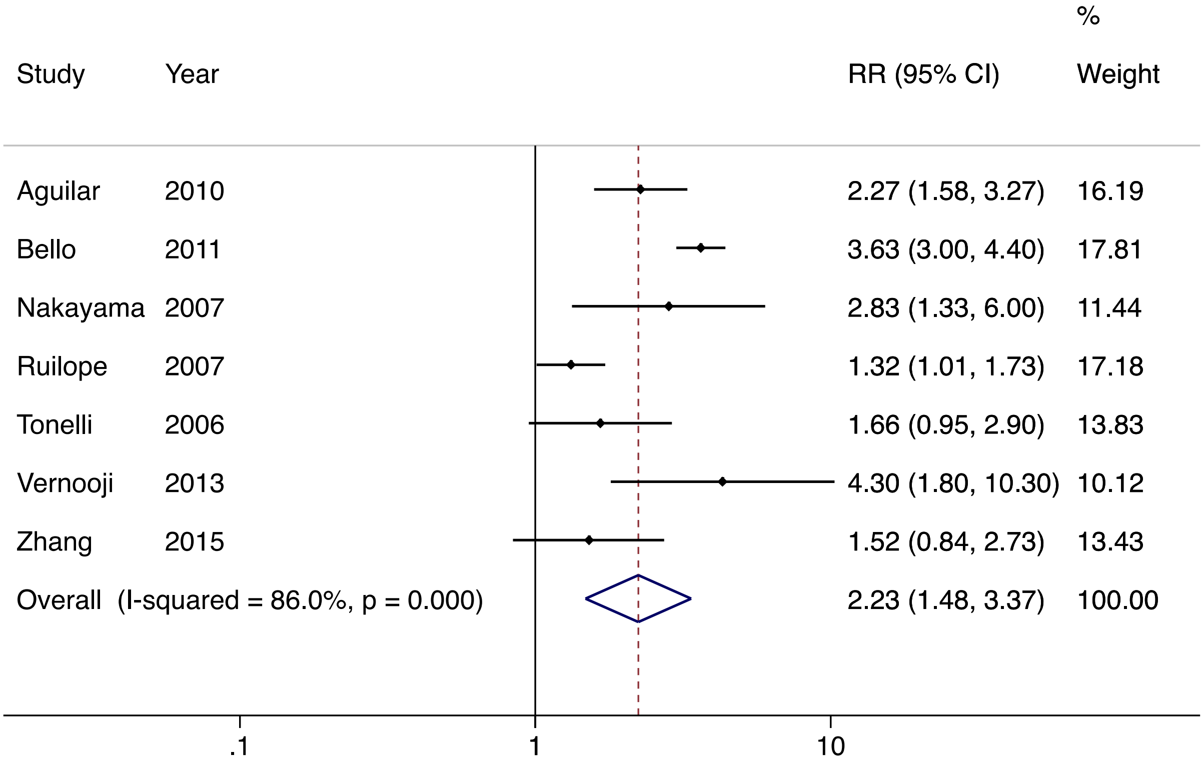

Appendix Figure VII: Funnel plot evaluating potential systematic bias in studies included in the meta-analysis

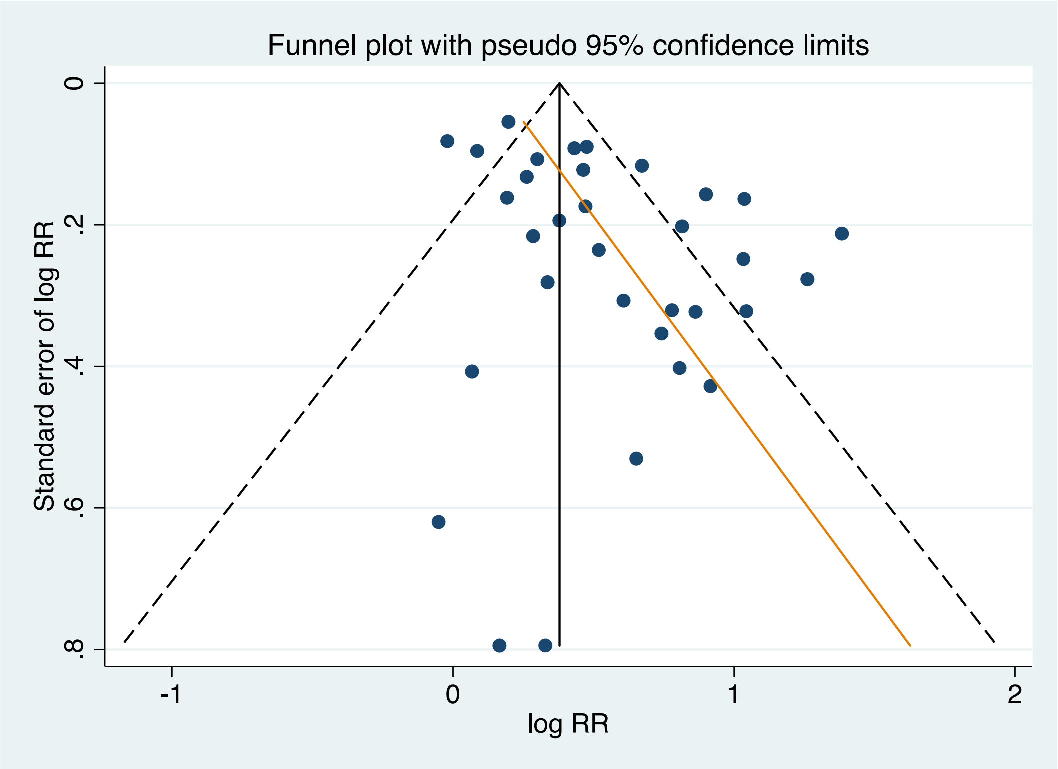

Appendix Table VI. Studies categorized according to a hierarchy of hypertension adjustment, from least (1) to best (4) adjustment

**1 = Baseline BP at study entry**	**2= History of HTN and/or on treatment and/or baseline BP at study entry**	**3= History of HTN and/or on treatment**	**4 = Multiple BP readings over time***
Hagg 2013<br>Hitman 2007<br>Irie 2006<br>Menne 2014<br>Nakayama 1997<br>Sander 2012<br>Sandsmark 2015<br>Tonelli 2006<br>Wagener 1994<br>Yang 2008	Fuller 2001<br>Lee 2016<br>Li 2015<br>Madison 2006<br>Miettinen 1996<br>Muntner 2012<br>Nagai 2014<br>Nakayama 2007<br>Yuyun 2004<br>Zang 2008	Bello 2011<br>Go 2009<br>McAlister 2017<br>Valmadrid 2000	Aguilar 2010<br>Da Costa 2017<br>De Leeuw 2002<br>Zhang 2015

** Including 24-hour ABPM*
